# Noninvasive Assessment of Antenatal Hydronephrosis in Mice Reveals a Critical Role for *Robo2* in Maintaining Anti-Reflux Mechanism

**DOI:** 10.1371/journal.pone.0024763

**Published:** 2011-09-20

**Authors:** Hang Wang, Qinggang Li, Juan Liu, Cathy Mendelsohn, David J. Salant, Weining Lu

**Affiliations:** 1 Renal Section, Department of Medicine, Boston University Medical Center, Boston, Massachusetts, United States of America; 2 Department of Urology, Columbia University, New York, New York, United States of America; 3 Department of Urology, Zhongshan Hospital, Fudan University, Shanghai, China; 4 Department of Nephrology, PLA General Hospital, Beijing, China; Childrens Hospital Los Angeles, United States of America

## Abstract

Antenatal hydronephrosis and vesicoureteral reflux (VUR) are common renal tract birth defects. We recently showed that disruption of the *Robo2* gene is associated with VUR in humans and antenatal hydronephrosis in knockout mice. However, the natural history, causal relationship and developmental origins of these clinical conditions remain largely unclear. Although the hydronephrosis phenotype in *Robo2* knockout mice has been attributed to the coexistence of ureteral reflux and obstruction in the same mice, this hypothesis has not been tested experimentally. Here we used noninvasive high-resolution micro-ultrasonography and pathological analysis to follow the progression of antenatal hydronephrosis in individual *Robo2*-deficient mice from embryo to adulthood. We found that hydronephrosis progressed continuously after birth with no spontaneous resolution. With the use of a microbubble ultrasound contrast agent and ultrasound-guided percutaneous aspiration, we demonstrated that antenatal hydronephrosis in *Robo2*-deficient mice is caused by high-grade VUR resulting from a dilated and incompetent ureterovesical junction rather than ureteral obstruction. We further documented *Robo2* expression around the developing ureterovesical junction and identified early dilatation of ureteral orifice structures as a potential fetal origin of antenatal hydronephrosis and VUR. Our results thus demonstrate that *Robo2* is crucial for the formation of a normal ureteral orifice and for the maintenance of an effective anti-reflux mechanism. This study also establishes a reproducible genetic mouse model of progressive antenatal hydronephrosis and primary high-grade VUR.

## Introduction

About one percent of human fetuses have congenital anomalies of the kidney and urinary tract (CAKUT), a family of birth defects comprising kidney anomalies such as antenatal hydronephrosis, duplex kidney and renal dysplasia, and ureteric defects such as vesicoureteral reflux (VUR) and urinary obstruction [1]. Although CAKUT is a complex genetically heterogeneous developmental disorder with variable phenotype, it can be caused by mutations in a single gene that controls early kidney and urinary tract development [2], [3], [4]. Antenatal hydronephrosis occurs in more than 1% of all pregnancies and is one of the most common CAKUT abnormalities detected on prenatal ultrasonography [5]. Antenatal hydronephrosis can be caused by a wide spectrum of renal and urological conditions ranging from transient dilatation of the fetal kidney collecting system that resolves spontaneously after birth to clinically significant urinary tract obstruction or VUR that leads to renal failure [6], [7]. As many as 1% children have VUR characterized by the retrograde flow of urine from the bladder to the ureter and kidney [8]. VUR is also the cause of 15–20% of cases of antenatal hydronephrosis [9], [10], [11]. Despite the high incidence of antenatal hydronephrosis and VUR in the pediatric population, the natural history, causal relationship, developmental origins and genetic basis of these clinical conditions remain ill-defined [12], [13], [14].

*ROBO2* is a member of the immunoglobulin superfamily and encodes a cell adhesion molecule involved in axonal guidance and neurogenesis [15], [16]. It is a receptor for SLIT2 ligand [17], and SLIT2-ROBO2 signaling acts as a chemorepulsive guidance cue to control axon pathfinding and neuron migration during nervous system development [15]. Slit2-Robo2 signaling is also crucial for normal development of the kidney and urinary collecting system. Expression of *Robo2* in the metanephric mesenchyme and *Slit2* in the ureteric bud is necessary for normal ureteric budding and reciprocal induction of nephrogenesis. Thus, mouse knockouts that lack *Slit2* or *Robo2* develop supernumerary ureteric buds, duplex kidneys, hydroureter and renal dysplasia [4], [18]. Recently, we and others have also found that human *ROBO2* is disrupted in a VUR patient with a chromosome translocation [4], and point mutations of *ROBO2* have been identified in VUR patients from several unrelated families [4], [19].

Conventional *Robo2* homozygous knockout mice die within 48 hours after birth due to severe abnormalities of the urinary tract [18]. Therefore, we have studied a mouse model with a conditional *Robo2* floxed allele, *Robo2* ^flox^. The *Robo2* ^flox/flox^ homozygotes are viable, fertile and lack urinary tract abnormalities [4]. Using an *EIIa-Cre* transgene that has variable Cre activity in the early pre-implantation embryo [20], we generated a *Robo2* ^del5/del5^↔*Robo2* ^del5/flox^ mosaic mouse line with *Robo2* gene dosage below haploinsufficiency [4]. Most *Robo2* mosaic mutant mice develop antenatal hydronephrosis and abnormally located ureterovesical junctions (UVJs). However, it is not clear if these abnormally located UVJs lead to urinary obstruction or reflux, both of which elevate the pressure in the upper urinary tract and are the common causes of antenatal hydronephrosis. Although many die in the postnatal period due to severe bilateral CAKUT, the variable deletion of the floxed *Robo2* allele in these mosaic mice allows some of them to survive into adulthood because the disease affects only one side [4]. This model has enabled us to answer several questions regarding the natural history, causal relationship and developmental origins of antenatal hydronephrosis and VUR, and to distinguish experimentally between urinary obstruction and reflux as a cause for antenatal hydronephrosis in *Robo2*-deficient mice.

In the study reported here, we used noninvasive micro-ultrasonography and pathological analysis to determine the natural history of antenatal hydronephrosis in individual *Robo2*-deficient mice from embryo to adulthood. With the use of a microbubble ultrasound contrast agent and dye tracing, we observed that *Robo2*-deficient mice developed high-grade dilating VUR accompanied by wide-open incompetent ureterovesical junctions without obstruction. By ultrasound-guided percutaneous aspiration, we determined that unilateral hydronephrosis in *Robo2*-deficient mice was exacerbated by the retrograde flow of urine from the contralateral functional kidney through an enlarged ureterovesical junction. Further developmental studies revealed early congenital dilatation of the ureteral orifice in *Robo2*-deficient mice with antenatal hydronephrosis and VUR. Our data provide strong evidence that *Robo2* is critical for maintaining both active and passive anti-reflux mechanisms and loss of *Robo2* gene could cause progressive congenital hydronephrosis and high-grade dilating VUR with little chance of spontaneous resolution after birth. This study also establishes that complex renal tract birth defects like antenatal hydronephrosis and high-grade VUR can be effectively studied noninvasively *in vivo* in mice.

## Results

### Noninvasive evaluation of perinatal hydronephrosis in *Robo2* mutant mice

**Figure 1:**
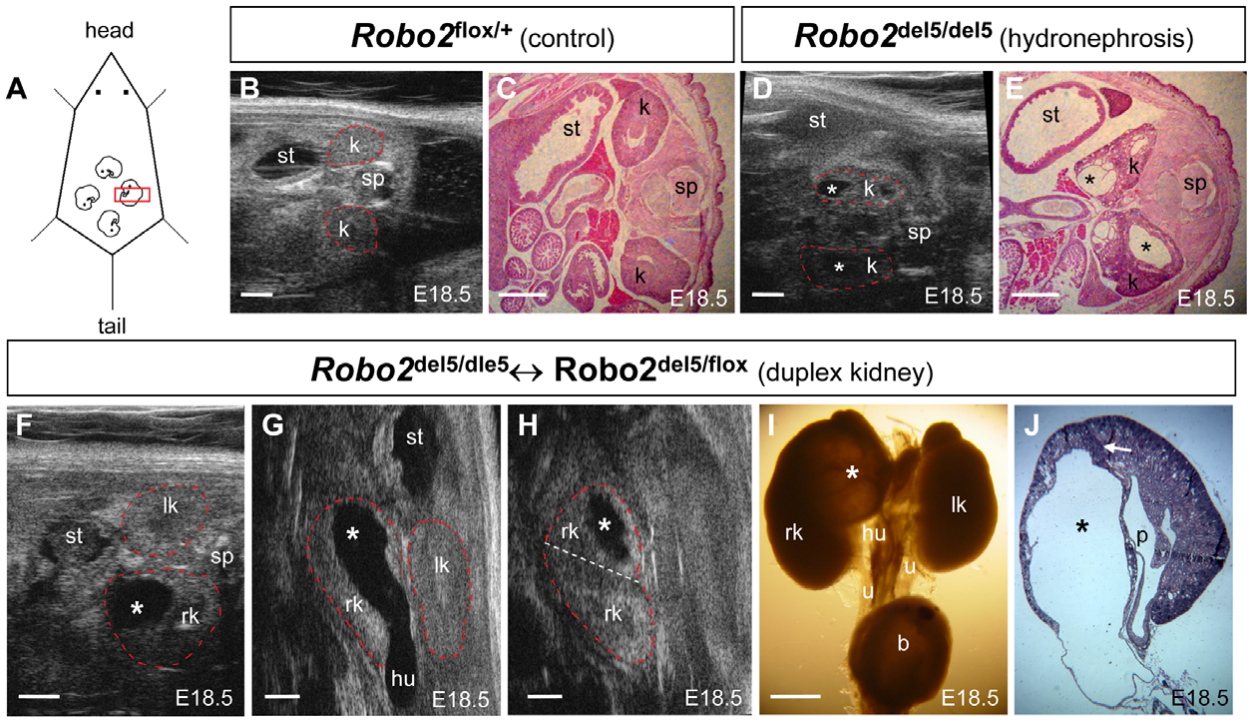
Noninvasive detection of antenatal hydronephrosis and duplex kidney in *Robo2* mouse embryos. The outline of the kidneys depicted in ultrasound images were marked by red dot line. (**A**) Schematic diagram of ultrasound detection of mouse embryonic kidney in pregnant mice; 4 schematic embryos inside a pregnant mouse were shown; red square represented the ultrasound scanhead. (**B**,**C**) Normal E18.5 mouse embryonic kidneys detected by Vevo 770 ultrasound (B) and H&E histology confirmation (C). (**D**,**E**) Ultrasound detection of antenatal hydronephrosis (asterisks) in an E18.5 *Robo2* homozygous embryo (D) and H&E histology confirmation (E) showing bilateral dysplastic kidney with hydronephrosis (asterisks). (**F**–**J**) Ultrasound detection of prenatal duplex kidney and upper pole hydronephrosis with hydroureter in an E18.5 *Robo2* mosaic embryo. Antenatal hydronephrosis (asterisks) in the upper pole of right kidney (rk) of an E18.5 *Robo2* mosaic embryo detected with B-mode ultrasound in transverse plane (F), ventral coronal plane (G) and dorsal coronal plane (H). A normal left kidney (lk) was also detected in the transverse plane (F) and ventral coronal plane (G). The hydronephrosis (asterisks) in the upper pole of right kidney (rk) was connected to an enlarged hydroureter (hu) in (G). A dorsal coronal plane (H) showing upper and lower right duplex kidney (divided by an artificial white dot line) as well as upper pole hydronephrosis (asterisks). (I) Gross structure of the urinary tract system from the same E18.5 *Robo2* mosaic embryo examined by ultrasound in (F–H) showing enlarged right duplex kidney with upper pole hydronephrosis (asterisks) connected to an enlarged hydroureter (hu). The right duplex kidney and upper pole hydronephrosis (asterisks) in this E18.5 *Robo2* mosaic embryo was confirmed by H&E histology (J); a white arrow depicting internalized nephrogenic zone (renal cortex) separating upper and lower duplex right kidney. Scale bars, 1.0 mm. Abbreviation: b, urinary bladder; k, kidney; lk, left kidney; rk, right kidney; p, renal pelvis; sp, spine; st, stomach.

In order to study the natural history of antenatal hydronephrosis in *Robo2*-deficient mice, we first established a noninvasive method to evaluate renal collecting system in mouse embryos. The human metanephric kidney begins to form at 4 weeks of gestation and is functional at approximately 11 gestational weeks, which corresponds to embryonic stages E15.5–E16.5 in mice. Using the transvaginal route [21] or Doppler imaging [22], a detailed evaluation of human embryonic kidney and renal pelvis by ultrasound is possible as early as 12 weeks [23]. To determine if ultrasonography can visualize mouse embryonic kidney at a comparable gestational age, we used a Visualsonic Vevo 770 high-frequency micro-ultrasound system to examine timed pregnant female mice (Fig. 1A and Figure S1A). The mouse fetal kidney could be detected by transabdominal ultrasonography as early as E15.5 when it started to produce urine (Figure S1), however the pelvis and ureters of normal wild-type and *Robo2* mutant embryos were indistinguishable at this stage (Figure S1B and S1C).

With rapid maturation of mouse embryonic kidney and urinary tract from E16.5 to birth, bilateral and unilateral antenatal hydronephrosis and duplex kidneys in *Robo2* mutant embryos could be diagnosed starting at E17.5–18.5 (Fig. 1B–J). We analyzed four E18.5 pregnant mice from *Robo2* heterozygous matings (Figure S2). Twenty-seven embryos were detected using Vevo 770 micro-ultrasound. Among these 27 embryos, 13 were diagnosed with antenatal hydronephrosis based on renal pelvic dilatation (Fig. 1D,F). After dissection, we recovered 29 E18.5 embryos and confirmed the presence of hydronephrosis in 13 embryos by histology (Fig. 1C,E,J). There were two false positives and two false negatives for the diagnosis of antenatal hydronephrosis with this micro-ultrasound method, which corresponds to 84.6% sensitivity and 87.5% specificity (Table S1).

**Figure 2:**
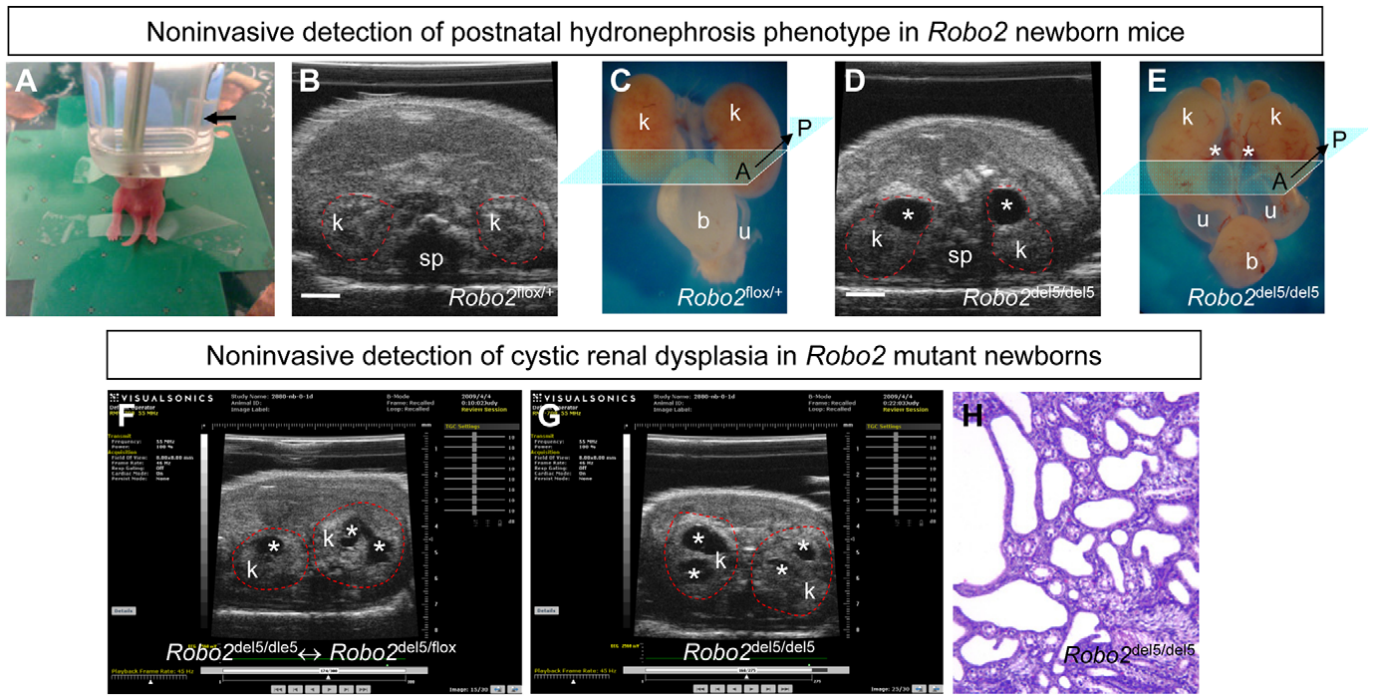
Noninvasive detection of postnatal hydronephrosis and cystic renal dysplasia phenotype in *Robo2* newborn mice. (**A**) Ultrasound scanhead position (arrow) and a newborn mouse under anesthesia. (**B**,**C**) Representative ultrasound and gross structure images of wild-type normal newborn kidneys. (**D**,**E**) Representative ultrasound and gross structure images of *Robo2* homozygous newborn kidneys with hydronephrosis (asterisks); an ideographic anterior to posterior (A→P) plane in (C,E) depicting B-mode ultrasound scanning. (**F**–**H**) Cystic renal dysplasia marked by multiple hypoechoic dark area (asterisks) were shown in a *Robo2* mosaic newborn (F) and a homozygous newborn (G). H&E histology of cystic renal dysplasia from a *Robo2* homozygous newborn was showed in (H). Scale bars, 1.0 mm. Abbreviation: b, urinary bladder; k, kidney; sp, spine; u, ureter.

We then analyzed newborn mice from six *Robo2* heterozygous matings when matched individual dissection and histological examination became possible. All 13 *Robo2* ^del5/del5^ homozygous newborn mice had hydronephrosis on micro-ultrasonography, which was confirmed by histology. Of 12 *Robo2* ^del5/del5^↔*Robo2* ^del5/flox^ mosaic newborn mice, eight (67%) had hydronephrosis detected by micro-ultrasonography and confirmed histologically (Fig. 2). Thus, micro-ultrasonography is 100% sensitive and 100% specific for detecting hydronephrosis in neonatal mice (Table S2). Interestingly, the majority (5/8, 63%) of *Robo2* ^del5/del5^↔*Robo2* ^del5/flox^ mosaic newborn mice with hydronephrosis displayed similar severity of hydronephrosis and cystic renal dysplasia phenotype as *Robo2* ^del5/del5^ homozygotes (Fig. 2F–H). This finding suggests that *Robo2* ^del5/del5^↔*Robo2* ^del5/flox^ mosaic mice can develop severe *Robo2*-null like hydronephrosis defects. Since *Robo2* ^del5/del5^↔*Robo2* ^del5/flox^ mosaic mice have variable ratios of the *Robo2* ^flox^ and the *Robo2* ^del5^ alleles due to the incomplete, stochastic action of the *EIIa-Cre* transgene on the *Robo2* ^flox^ alleles in the early embryo before implantation [4], [20], some mosaic mice also survive to adulthood with relatively mild hydronephrosis [4]. Therefore, the mosaic *Robo2* genotype alone cannot predict the severity of hydronephrosis, and variable *Robo2* gene dosage may be responsible for the broad-spectrum and variable severity of the CAKUT phenotype in *Robo2* ^del5/del5^↔*Robo2* ^del5/flox^ mosaic mice. Of note, a similarly broad-spectrum and variable severity of urinary tract abnormalities has also been found in human CAKUT patients [24], [25]. Thus, these studies establish that micro-ultrasonography is a sensitive and reliable method for noninvasive *in vivo* investigation of complex renal and urological birth defects like CAKUT in embryonic and newborn mice.

### Antenatal hydronephrosis in *Robo2* knockout mice progresses continuously without spontaneous resolution after birth

Compared with the prenatal diagnosis of hydronephrosis in humans, a challenge in mouse fetal ultrasound examination is the large number of mouse embryos in each pregnancy with an average litter size of eight fetuses. To study the natural history of antenatal hydronephrosis, it is important to identify individual embryos with hydronephrosis prenatally and follow its disease progression after birth. Although it is difficult to distinguish individual normal embryos because their anatomies are similar, by monitoring hydronephrosis severity, symmetry and position of the urinary tract, we have successfully identified and followed E18.5 mutant embryos from eight pregnant mice of *Robo2* heterozygous mice matings (Figure S2). Using Vevo 770 micro-ultrasound, we matched individual embryos and newborn mice with the same characteristic urinary tract structural abnormalities such as hydronephrosis, hydroureter and duplex kidney (Figure S3). This procedure enabled us to follow the antenatal hydronephrosis progression noninvasively in the same *Robo2*-deficient mouse from embryo to neonate, a period with major physiological and functional changes during kidney and urinary tract development.

**Figure 3:**
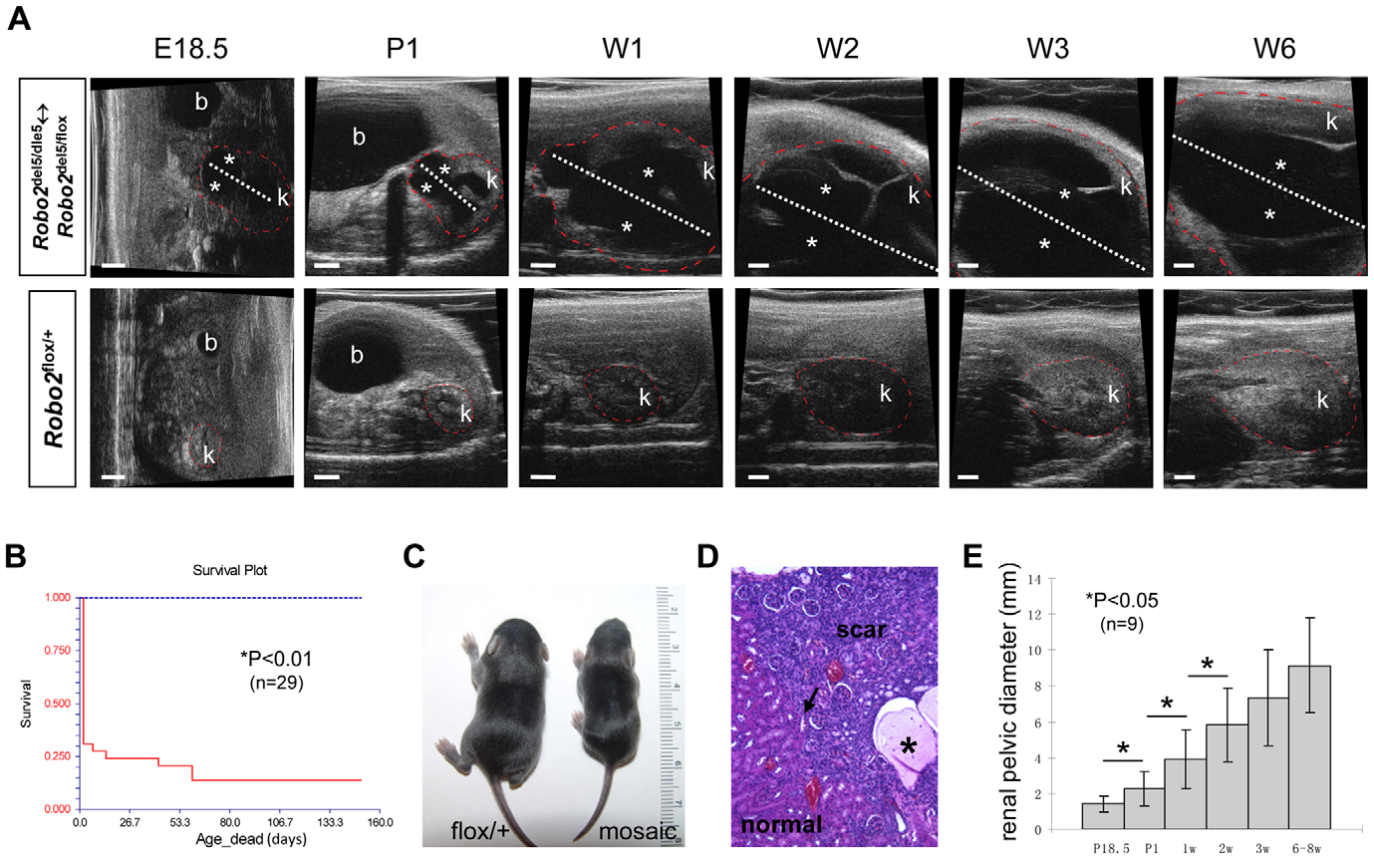
Natural history of antenatal hydronephrosis in *Robo2* mosaic mice. (**A**) Upper figure panels: ultrasound noninvasive monitoring of hydronephrosis (asterisks) progression in a *Robo2* mosaic mutant mouse from embryonic E18.5 to postnatal 6-week old (W6) showing no spontaneous resolution; white dot line representing the anteroposterior renal pelvic diameter. Lower figure panels: ultrasound noninvasive monitoring of a wild-type littermate control showing absence of hydronephrosis at any stages. The outline of the kidneys depicted in ultrasound images have been marked by red dot line. P1: postnatal day-1; W1 to W6: week-1 to week-6; b: bladder; k: kidney. (**B**) Survival curve of *Robo2* mice with antenatal hydronephrosis (red line) compared with the survival curve of *Robo2* mice without antenatal hydronephrosis (blue line). (**C**) Small body size of a *Robo2* mosaic mouse with hydronephrosis at one week compared with its age matched wild-type littermate. (**D**) H&E histology of nephropathy and renal scar in a *Robo2* mosaic mouse showing atrophic tubules, substantial inflammatory cellular infiltrate, degeneration of remnant glomeruli and proteinaceous casts (asterisk); there was a reflux nephropathy characteristic abrupt transition from scar tissue to relatively normal parenchyma (arrow in D). (**E**) The severity of hydronephrosis measured by the anteroposterior renal pelvic diameter at different stages from prenatal E18.5 to postnatal week-6 showing continuous progression of hydronephrosis in *Robo2* mice without spontaneous resolution.

To determine the natural history of disease progression of antenatal hydronephrosis in *Robo2* mice, we identified 67 E18.5 embryos from the eight pregnant mice of *Robo2* heterozygous matings noted above and followed them to postnatal week six by ultrasound (Fig. 3). The anteroposterior renal pelvic diameter of each mouse was used as a marker to measure progression of hydronephrosis (Fig. 3A). Among these 67 E18.5 embryos, 29 (43%) developed antenatal hydronephrosis, and of these, 25 (37%) were bilateral and four (6%) were unilateral. Three (5%) embryos had a duplex kidney with upper pole hydronephrosis (one bilateral and two unilateral), and five (7%) embryos developed combined unilateral hydronephrosis and contralateral duplex kidney (with upper pole hydronephrosis). After birth, 20 (80%) mice with bilateral antenatal hydronephrosis died within 48 hours (Fig. 3B and Table S3). Only two (8%) mice with bilateral antenatal hydronephrosis survived to 6 weeks and died in week 9 with small body size (Fig. 3C) and renal scarring suggesting reflux nephropathy (Fig. 3D). However, all four (100%) mice with unilateral antenatal hydronephrosis survived (Table S3) together with three (100%) mice with duplex kidney and upper pole hydronephrosis. We then monitored the renal pelvic diameters of seven surviving mice with antenatal hydronephrosis (two bilateral, four unilateral, one with combined antenatal hydronephrosis and duplex kidney – total nine kidney units with antenatal hydronephrosis) to postnatal week 6. The degree of hydronephrosis progressed rapidly with no spontaneous resolution (Fig. 3A) and the most significant enlargement of renal pelvis occurred in the first two weeks of postnatal life (Fig. 3E and Table S4). Since mouse nephrogenesis does not cease until 2 weeks after birth, this result supports the idea that severe hydronephrosis is especially detrimental to the developing kidney [26], [27]. Interestingly, micro-ultrasound follow-up of three mice with duplex kidney (one bilateral and two unilateral – total four duplex kidney units with upper pole hydronephrosis) showed spontaneous resolution of the upper pole hydronephrosis (Figure S4). These results suggest that severe progressive antenatal hydronephrosis caused by *Robo2* mutations is a significant risk factor for postnatal death.

### Hydronephrosis in *Robo2* mutant mice is caused by ureterovesical junction dilatation and vesicoureteral reflux

**Figure 4:**
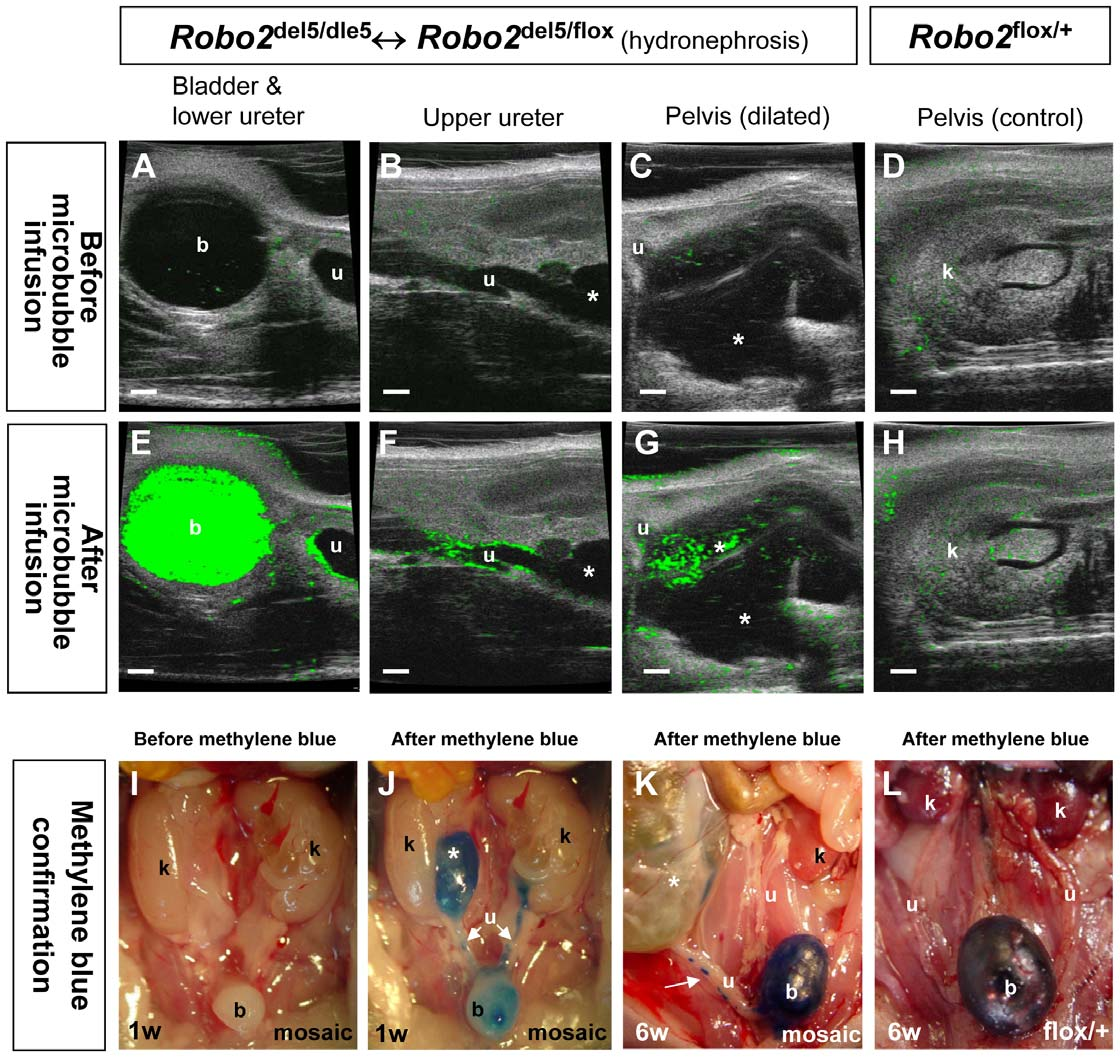
Hydronephrosis in *Robo2* mosaic mice is caused by high-grade VUR. (**A**–**D**) Ultrasonographic images (green-scale under contrast-mode) depicted the urinary tract system and hydronephrosis (asterisk in C) in a *Robo2* mosaic mouse (A–C) and the kidney pelvis region of a *Robo2* ^flox/+^ control (D) before infusion of microbubble ultrasound contrast agent into the mouse bladder. Some background green signals were also seen in the green-scale setting. (**E**–**G**) Ultrasonographic images (green-scale under contrast-mode) after microbubble contrast agent infusion showing the flow of microbubble (strong and fresh green signals) inside the same urinary tract system of the *Robo2* mosaic mouse after infusion of microbubble into the bladder. The green color enhanced microbubble was refluxed into the renal pelvis (asterisks in G; also see Video S3) through the ureter (F). Only background green signals were seen in the *Robo2* ^flox/+^ control (**H**) without reflux. (**I**–**K**) Methylene blue cystogram was used to confirm reflux phenotype in an one week old *Robo2* mosaic mouse (I,J) and a six weeks old *Robo2* mosaic mutant mouse (K) showing retrograde flow of methylene blue into the renal pelvis (asterisks) through the ureter (white arrows in J,K). (**L**) A six weeks old wild-type control with methylene blue inside the bladder without reflux. Scale bars, 1.0 mm. Abbreviation: b, urinary bladder; k, kidney; u, ureter.

The phenotype of antenatal hydronephrosis and nephropathy can be caused by either urinary tract obstruction or reflux, both of which elevate the pressure in the upper urinary tract. We have previously showed that *Robo2* mosaic mice developed abnormally located ureterovesical junctions (UVJs) [4]. To determine if these abnormally located UVJs in *Robo2*-deficient mice lead to VUR or urinary obstruction, we performed noninvasive echo-enhanced ultrasonic cystography with a microbubble ultrasound contrast agent (UCA) in four 6-week old *Robo2* female mosaic mice that had been diagnosed with unilateral antenatal hydronephrosis and four age-matched wild-type controls. The reason to choose female mice for this experiment is a technical one since there is a low success rate of urethral catheterization in adult male mice [28]. We and others have recently tested the feasibility of this microbubble method in inbred wild-type mice with low-grade non-dilating VUR without hydronephrosis by 3D reconstruction [29]. In order to determine if *Robo2* mosaic mice developed high-grade dilating VUR with free flow of microbubble inside the mouse urinary tract without 3D reconstruction, we used the innovative FunnelCath urethral catheter for mice. This infusion friendly mouse catheter has a thin distal segment for easy entry into the mouse urethra and can be visualized inside the mouse bladder by ultrasonic scan (Figure S5). After infusion of Option (a FDA-approved microbubble UCA) into the urinary bladder (Fig. 4, Video S1 and Video S2), we found that all (4/4, 100%) *Robo2* mosaic mutant mice with hydronephrosis displayed VUR (Fig. 4A–H, Video S3). Sequential images showed that the microbubble UCA flowed retrogradely from the bladder into the dilated ureter (Fig. 4F) and was seen in the renal pelvis (Fig. 4G, Video S3) about 3 seconds after the infusion of the microbubble UCA into the bladder, which suggests a low pressure reflux. The microbubble UCA was washed out from renal pelvis quickly (as fast as 18 seconds) after it appeared (Figure S6, Video S4), suggesting that no obstruction was present in the upper urinary tract of *Robo2* mutant mice. None of four (4/4, 100%) control mice displayed urinary reflux after Option microbubble infusion into the bladder (Fig. 4H). The reflux phenotype was confirmed in *Robo2* mosaic mice by infusing methylene blue into the bladder (Fig. 4I–L), an open abdominal invasive method that is the current gold standard to evaluate VUR in mice [30], [31]. These results indicate that loss of *Robo2* indeed causes urinary reflux rather than urinary obstruction as previously thought [4] and the *Robo2* mosaic mouse represents an excellent animal model for congenital high-grade dilating VUR.

**Figure 5:**
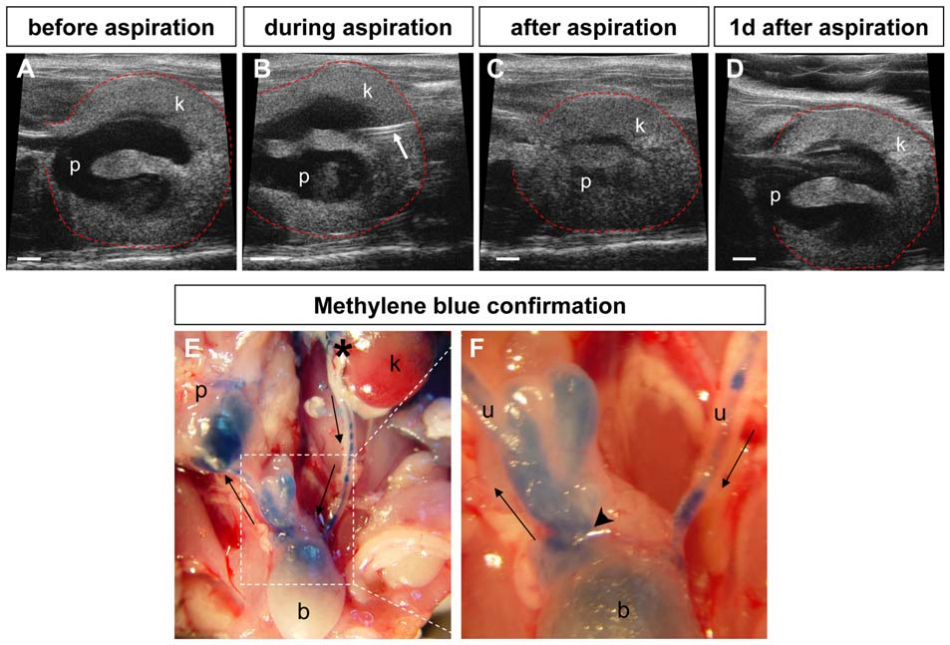
Causal relationship of hydronephrosis and VUR in *Robo2* mosaic mice. Unilateral hydronephrosis were exacerbated by the retrograde flow of urine from the contralateral functional kidney. (**A**–**D**) Ultrasound-guided percutaneous aspiration of urine from the right renal pelvis of a *Robo2* mosaic mouse with severe unilateral hydronephrosis: (A) right renal pelvis with hydronephrosis before ultrasound-guided needle insertion; (B) ultrasound-guided needle (arrow) insertion; (C) collapsed right renal pelvis after ultrasound-guided needle aspiration; (D) reappeared right hydronephrosis one-day after aspiration. p, pelvis; k, kidney. (**E**,**F**) Methylene blue dye tracing showing retrograde flow of urine refluxing into the right renal pelvis from contralateral functional left kidney after methylene blue injection into the left renal pelvis; asterisk in (E) marked the methylene blue injection site in the left renal pelvis; arrows indicated the direction of urine flow that was labeled by methylene blue. (F) High magnification of boxed ureterovesical junction region in panel (E) showing abnormal enlarged right ureteral orifice (arrowhead) which disrupted the anti-reflux mechanism and allowed ureteral reflux to occur.

To determine if there is a causal relationship between VUR and hydronephrosis and if the process of hydronephrosis formation in *Robo2* adult mice is active or a residual effect of antenatal hydronephrosis, we performed ultrasound-guided percutaneous aspiration of the unilateral hydronephrotic kidney in three *Robo2* mosaic mutant mice (Fig. 5A–C, Video S5 and Video S6). Interestingly, we noted rapid re-accumulation of urine in the renal pelvis within 24 hours (Fig. 5D). To determine the source of the urine that reconstituted the unilateral hydronephrosis, we injected methylene blue into the renal pelvis of the contralateral unaffected kidney (Fig. 5E) and found that urine flowed into the dilated renal pelvis through the bladder and enlarged ureterovesical junction (Fig. 5E,F). In addition to further demonstrating the VUR phenotype, these results suggest that unilateral hydronephrosis in *Robo2* mice is exacerbated by the retrograde flow of urine from the contralateral functional kidney through an enlarged ureterovesical junction, which may further impair the growth, development and function of an already dysplastic kidney.

### Fetal origin of antenatal hydronephrosis and VUR phenotype in *Robo2* mutant mice

**Figure 6:**
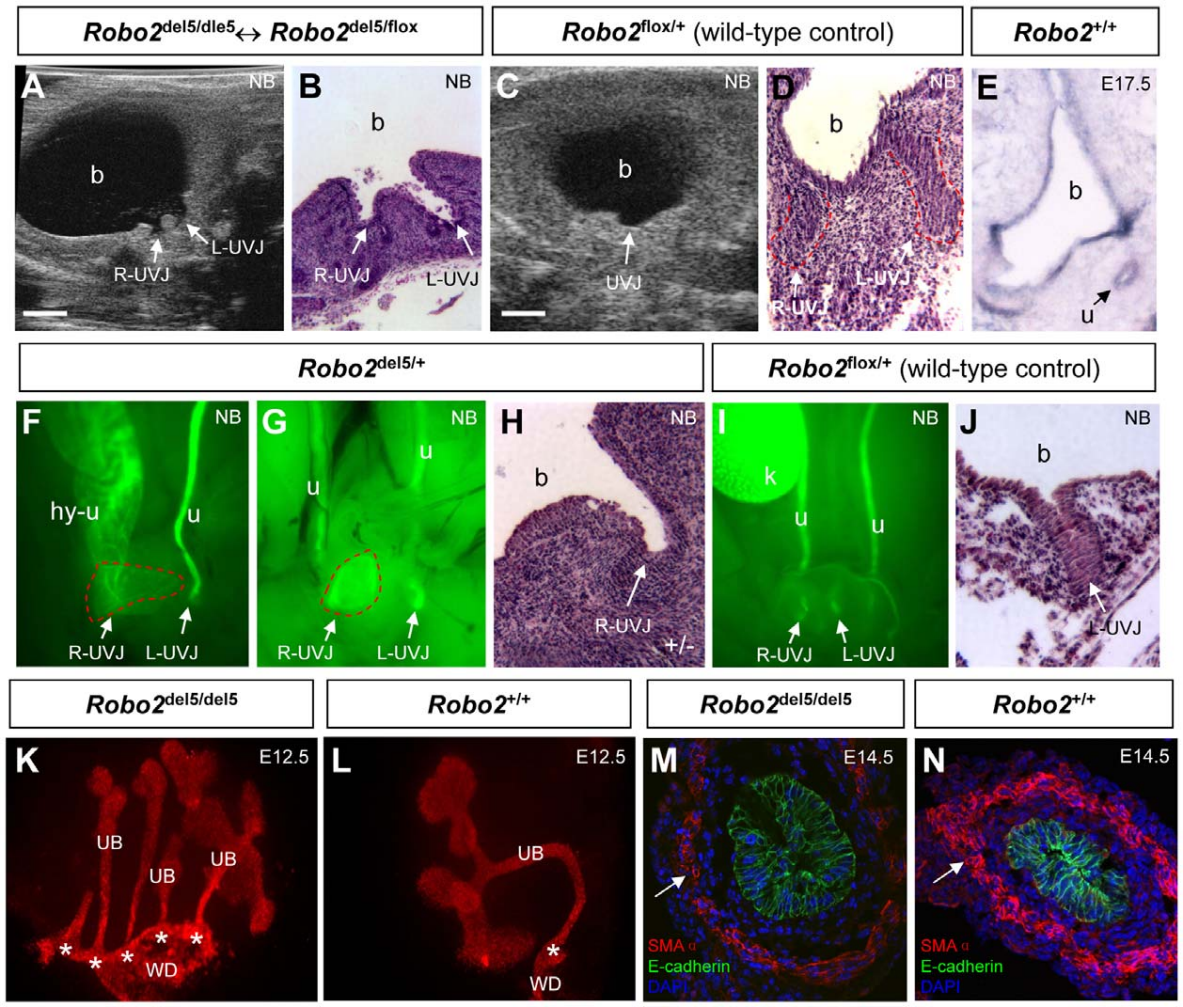
Fetal origin of antenatal hydronephrosis and VUR phenotype in *Robo2* mutant mice. (**A**) Noninvasive *in vivo* ultrasound detection of bilateral golf-hole like ureterovesical junction (UVJ) dilatation (arrows) in a newborn *Robo2* mosaic mouse (also see Video S7); Scale bar, 1.0 mm; R-UVJ: right UVJ; L-UVJ: left UVJ; b: bladder. (**B**) H&E histology of the same newborn mice in (A) confirmed the bilateral golf-hole like UVJ dilatation (arrows); Magnification: 100×. (**C**) An un-dilated normal UVJ (arrow) was barely detectable under ultrasound scan in a wild-type control newborn mouse; Scale bar, 1.0 mm. (**D**) H&E histology of the same control newborn mice in (C) showing un-dilated right and left UVJs outlined by red dot line; Magnification: 100×. (**E**) *In situ* hybridization analysis showing *Robo2* transcripts expressed around the developing UVJ: in the distal ureter (u, arrow) inside the bladder (b) wall of an E17.5 mouse embryo; Magnification: 50×. (**F**–**H**) Unilateral UVJ dilatation in *Robo2* heterozygous newborn mice: (F,G) Hoxb7-GFP reporter gene positive *Robo2* newborn heterozygotes showing unilateral right UVJ dilatation (red dot line in panel F,G), associated with right hydroureter (hy-u in panel F) or undilated ureter (u in panel G); the left UVJ in *Robo2* newborn heterozygotes (L-UVJ in panel F and G) appeared normal; Magnification: 25×. (H) H&E histology confirmed right UVJ dilatation (arrow) in a *Robo2* newborn heterozygote; Magnification: 100×. (**I**,**J**) Both right and left UVJ (arrows) of a wild-type newborn control appeared normal by Hoxb7-GFP (panel I) or H&E histology (panel J) analyses; Magnification: 25× for panel I, 100× for panel J. (**K**) Early multiple ureteric buds (UB) outgrowth from the Wolffian duct (WD) in a *Robo2* ^del5/del5^ homozygous embryo (E11.5 kidney cultured for 1-day and stained with pan-cytokeratin); magnification: 60×. A wild-type embryo in (**L**) showing a single UB with one budding site (asterisk) from the Wolffian duct (WD); magnification: 60×. (**M**,**N**) Anti-smooth muscle α actin antibody (SMAα, red) showing reduced and irregular ureteral smooth muscle staining (arrow in M) in an E14.5 *Robo2* ^del5/del5^ homozygous embryo compared with its wild-type littermate control (arrow in N). The urothelium (stained by anti-E-cadherin antibody, green) appeared normal in both *Robo2* ^del5/del5^ homozygous and wild-type controls; DAPI (blue) marked cell nuclei; Magnification: 400×.

To determine if an enlarged ureterovesical junction was the primary congenital defect at an early developmental stage in *Robo2* mutant embryos and later caused reflux and hydronephrosis, we examined the structure of the ureterovesical junction and entire upper urinary tract by micro-ultrasonography in 30 *Robo2* newborn mice with antenatal hydronephrosis (Video S7 and Video S8). Interestingly, 25 (83%) of 30 *Robo2* newborn mutant mice with hydronephrosis displayed wide open golf-hole like ureterovesical junctions (Fig. 6A, Video S7 and Video S8) and were confirmed by histology (Fig. 6B). The ureterovesical junction structure was barely detectable by ultrasound in a cohort of 30 (30/30, 100%) control newborn mice without hydronephrosis (Fig. 6C,D). To determine if *Robo2* and its ligand *Slit2* have intrinsic roles and are expressed in the ureterovesical junction region during development, we performed *in situ* hybridization and found both *Robo2* and *Slit2* mRNA in the distal ureter inside the bladder wall of developing mouse embryos (Fig. 6E and Figure S7). These results suggest that *Robo2* is required for normal ureterovesical junction formation and that loss of *Robo2* can lead to early congenital ureterovesical junction dilatation, which disrupts the anti-reflux mechanism in the bladder trigone.

To determine if ureterovesical junction dilatation is an early developmental defect in *Robo2*-deficient mice, we utilized the expression of the *Hoxb7-GFP* transgene, an excellent marker of the ureteric bud and its derivatives from E10.5 on [32]. Since the *Hoxb7-GFP* transgene is allelic to *Robo2* ^flox^ or *Robo2* ^del5^ allele, this precluded its use in *Robo2* ^del5/del5^ homozygotes and *Robo2* ^del5/del5^↔*Robo2* ^del5/flox^ mosaic mice. However, we found three newborn *Robo2* ^del5/+^ heterozygous mice with unilateral dilatation of ureterovesical junction (Fig. 6F,G), two of which had a dilated ureter on the same side (Fig. 6F). This unilateral ureterovesical junction dilatation was confirmed by histology (Fig. 6H) and was not found in 20 wild-type newborn controls (Fig. 6I,J). Since formation of the normal ureterovesical junction during early embryonic development is critically dependent on the primordial ureteric budding site from the Wolffian duct [33], we examined the ureteric bud outgrowth in early *Robo2* ^del5/del5^ homozygous embryos. By pan-cytokeratin staining (another marker for ureteric bud and its derivatives) of five cultured kidney explants, we found supernumerary ureteric buds in all *Robo2* ^del5/del5^ homozygous embryos (Fig. 6K), but not in wild-type controls (Fig. 6L). Because normal ureteral smooth muscle development is critical for ureterovesical junction formation and bladder trigone structure [34], we next examined the morphology of ureteral smooth muscle in early *Robo2* ^del5/del5^ homozygous embryos before ureter dilatation by triple and dual immunohistochemistry staining. Using anti-smooth muscle α actin antibody (a ureter smooth muscle marker), E-cadherin (a marker for ureter urothelium) and lectin *Dolichos Biflorus* (DBA, another marker for ureter urothelium), we found normal looking urothelium but disarrangement and reduced abundance of ureteral smooth muscle in three E14.5 *Robo2* ^del5/del5^ homozygous embryos (Fig. 6M,N and Figure S8) before any ureteral dilatation or hydronephrosis. These results suggest that Robo2 signaling may be necessary for the intercalation of ureteric smooth muscle into the bladder trigone to form a normal ureterovesical junction [34].

## Discussion

The ureter normally enters the bladder in an oblique direction, perforating the bladder muscle (detrusor) laterally and proceeding between the bladder mucosa and detrusor muscle to form the ureterovesical junction (UVJ) also called ureteral orifice. The intravesical portion of the ureter collapses during bladder voiding and creates a “passive” so-called flap-valve mechanism that prevents reflux [35], [36]. However, recent studies show that the ratio of the intravesical ureter length to the ureter diameter is smaller than previously thought [37], and suggest that there is also an “active” ostial-valve anti-reflux mechanism controlled by the contraction of the muscle coat around the ureterovesical junction [38], [39]. The muscle fiber of the ureterovesical junction functions as an “ureterovesical sphincter” that contracts in response to bladder contraction during voiding and subsequently relaxes following the closure of the external urethral sphincter during bladder filling [40], [41]. This active ostial-valve function of the ureterovesical junction allows coordinated opening and closure of the ureteral orifice, which represents a boundary area between the low pressure urinary segment in the upper urinary tract (i.e. the ureter and kidney) and the highly variable pressure segment in the lower urinary tract (i.e. the bladder and urethra). Together, the active and passive anti-reflux mechanisms act as a one-way valve allowing ejection of the bolus of urine into bladder lumen when its pressure is low and preventing retrograde urine reflux when the bladder pressure is high [40], [41].

Our present study provides strong evidence that *Robo2* is critical for maintaining both active and passive anti-reflux mechanisms. First, low pressure retrograde urine flow was clearly demonstrated in *Robo2* mutant mice via noninvasive microbubble contrast agent enhanced urosonography, a safe, reliable and radiation-free imaging modality that has been widely used in clinical medicine to detect VUR in children [42]. Second, both ultrasound-guided percutaneous aspiration and dye tracing studies demonstrated that postnatal *Robo2* mice with unilateral hydronephrosis had an incompetent anti-reflux mechanism and developed urinary reflux by the retrograde flow of urine from the contralateral functional kidney through enlarged ureteral orifice. In VUR patients, this condition is often referred to as vesicoureteral reflux dysplasia (VURD) syndrome [43], [44], [45]. The term of VURD was initially introduced to describe persistent unilateral reflux and renal dysplasia in boys with posterior urethral valves (PUVs) [44]. However, we have neither identified PUVs in *Robo2* mutant mice nor observed any signs of PUV-associated bladder outlet obstruction, which are characterized by bladder dilatation and bladder wall thickening with PUV-specific ultrasound finding (i.e. bladder neck keyhole sign) in male gender only [46]. Instead, most *Robo2* mutant mice developed golf-hole like ureteral orifice dilatation in both male and female gender with normal bladder volume and thin bladder wall. These results suggest that VURD may also happen in children with non-obstructive uropathy like primary dilating VUR. The third evidence supporting a role for *Robo2* in the anti-reflux mechanism is the golf-hole like ureteral orifice dilatation detected by both micro-ultrasonography and *Hoxb7-GFP* reporter gene in *Robo2* mutant mice with hydronephrosis. This ureteral orifice dilatation could disrupt both active and passive anti-reflux mechanisms and lead to urinary reflux. Since the dilatation of ureterovesical junction structure in *Robo2* mutant mice was too severe to be corrected by postnatal trigone or ureteral maturation and remodeling, we found no spontaneous resolution of hydronephrosis in *Robo2* mutant mice with congenital golf-hole like wide-open ureteral orifice detected at birth. Interestingly, similar ureterovesical junction defects of bilateral wide-open ureteral orifices and absence of intravesical ureteral segments have already been found in a patient with dilating VUR and *Robo2* gene disruption [4]. The golf-hole like ureteral orifice dilatation has also been recognized in high-grade dilating VUR patients with no spontaneous resolution [47]. Lastly, in addition to its expression in early metanephric mesenchyme, *Robo2* (and its ligand *Slit2*) is expressed in the developing ureterovesical junction. Malformed and disorganized ureteral muscle fiber architecture in *Robo2* homozygous mutant embryos could also lead to attenuation of the trigone and dysfunction of active valve mechanism of ureterovesical junction, which is formed by intercalation of ureteral muscle fibers and bladder muscle during development [34]. Similarly, the muscular wall of the distal ureter in VUR children is decreased and replaced with dysplastic and atrophic smooth muscle cells [38], [39], [48].

The inner layer of ureteral smooth muscle cells are derived from the early mesenchymal cells that surround the mesonephric duct [34], [49]. Since *Robo2* is specifically expressed in the early mesenchymal cells surrounding the mesonephric duct before ureteric bud outgrowth [18], it is not unlikely that loss of *Robo2* in early mesenchymal progenitor cells may lead to abnormal ureteral smooth muscle cell development, ureterovesical junction dilatation, trigonal weakness and VUR [50]. Because an early ectopic ureteric bud could not acquire adequate surrounding mesenchymal tissue to form proper trigonal musculature and ureterovesical junction, it is more prone to become a refluxing ureter later. Thus, multiple ureteric buds in *Robo2* mutant embryos could also cause weak ureteral musculature and gaping ureteral orifices with incompetent anti-reflux mechanism [33], [50]. Future studies are required to test these hypotheses and provide additional mechanistic insights into the role of Robo2 in the development of ureter smooth muscle cells and ureterovesical junction.

We have applied a micro-ultrasound system for noninvasive and longitudinal investigations of antenatal hydronephrosis and VUR in a genetically engineered mouse model of a recently identified human primary VUR disorder (VUR2, OMIM #610878). With this noninvasive imaging modality, we were able to study the natural history and disease progression of complex CAKUT birth defects in the same individual mouse from embryonic stage to adulthood. Our results showed that loss of *Robo2* gene could cause progressive congenital hydronephrosis and high-grade dilating VUR with little chance of spontaneous resolution after birth. Perinatal genetic analysis for *ROBO2* mutations might serve as an alternative to medical surveillance and watchful waiting, and may be used in the future as an early diagnostic tool to identify a subset of affected fetuses and newborns with progressive hydronephrosis and VUR. With this genetic testing, early ureterovesical junction surgery such as ureteral reimplantation might be planned for newborns with *ROBO2* mutations to minimize further renal injury caused by progressive reflux and preserve remaining renal function. In addition, our mouse model also informs us that we may uncover more deleterious mutations in *ROBO2* gene if we focus on DNA analysis of patients with both progressive antenatal hydronephrosis and congenital high-grade dilating VUR. Finally, our study also provides a reproducible genetic mouse model of congenital progressive antenatal hydronephrosis and primary dilating VUR, which will improve our understanding of the pathophysiology and disease process of these challenging conditions so that we may treat patients more efficiently [51].

## Materials and Methods

### Animals

#### Ethics Statement

Mouse protocols were approved by the Institutional Animal Care and Use Committee (IACUC) at Boston University Medical Center (#14388).

### Preparation of *Robo2* mutant mice for analysis

The generation and genotyping of *Robo2* ^flox^ conditional allele, *Robo2* ^del5^ germline mutant allele, *Robo2* ^+^ wild-type allele, and *Robo2* homozygous, heterozygous and mosaic mice were described previously [4]. Briefly, the *Robo2* heterozygous breeding scheme in Figure S2 was followed to generate F1 pregnant mice carrying embryos of *Robo2* ^del5/del5^ homozygotes, *Robo2* ^del5/flox^;*Tg* ^Ella-Cre+^ (*Robo2* ^del5/del5^↔*Robo2* ^del5/flox^) mosaics, *Robo2* ^del5/+^ heterozygous, and *Robo2* ^flox/+^ controls as well as newborn and adult mice in a mixed C57BL/6-129/Sv background. In *Robo2* ^del5/flox^;*Tg* ^Ella-Cre+^ mosaic mice, EIIa-Cre recombinase deletes only the *Robo2* ^flox^ allele to facilitate the penetrance of CAKUT phenotype when the *Robo2* gene dosage is reduced below haploinsufficiency (the other allele, *Robo2* ^del5^, has been deleted ubiquitously from germline expression). The *Robo2* mosaicism originates from the incomplete, stochastic action of the *EIIa-Cre* transgene on the *Robo2* ^flox^ alleles in the early embryo before implantation [4], [20]. This particular union of *Robo2* ^del5^;*Tg* ^Ella-Cre^ and *Robo2* ^flox^ gametes results in *Robo2* ^del5/del5^↔*Robo2* ^del5/flox^ mosaic mice. These mosaics are genotypically denoted *Robo2* ^del5/del5^↔*Robo2* ^del5/flox^ because they contain both *Robo2* ^del5/del5^ and *Robo2* ^del5/flox^ cells (*i.e.* a mixture of homozygous and heterozygous cells). Cells carrying the *Robo2* ^del5/del5^ genotype in these *Robo2* ^del5/del5^↔*Robo2* ^del5/flox^ mosaics derive from the action of *EIIa-Cre* in cells that commence embryogenesis with the *Robo2* ^del5/flox^ genotype. *Robo2* ^del5/flox^ cells remain when *Ella-Cre* activity in those cells is incomplete or insufficient to cause Cre-loxP mediated recombination. The authenticity of *Robo2* ^del5/flox^;*Tg* ^Ella-Cre+^ (*Robo2* ^del5/del5^↔*Robo2* ^del5/flox^) mosaic mice was determined by tail DNA genotyping for the presence of *Robo2* ^del5^ and *Robo2* ^flox^ alleles as well as *Tg* ^Ella-Cre^ transgene. F1 timed pregnant female mice (after positive vaginal plug) were examined by micro-ultrasound for prenatal CAKUT phenotype in mouse embryos. F2 *Robo2* ^del5/del5^ homozygous and *Robo2* ^del5/del5^↔*Robo2* ^del5/flox^ mosaic mice were examined by micro-ultrasound for postnatal CAKUT phenotype. F2 littermates *Robo2* ^flox/+^ mice without CAKUT phenotype were used as controls. To examine the ureterovesical junction defects in *Robo2* ^del5/+^ newborn mice, *Hoxb7-GFP* transgenic mice (a gift from Dr. Frank Costantini, Columbia University) were bred with *Robo2* ^del5/+^ mutants. GFP fluorescence was monitored and photographed using an Olympus SZX16 epifluorescence stereomicroscope.

### Micro-ultrasound analysis of prenatal CAKUT phenotype in mouse embryos

The Vevo 770 micro-ultrasound imaging system from VisualSonics (www.visualsonics.com) was used for all noninvasive ultrasound imaging analyses. Vevo 770 (VisualSonics, Toronto, Canada; #VS-11392) is a high frequency (25–55 MHz) micro-ultrasound system specially designed to assess anatomical structures and flow dynamics in small animals such as mice. It provides spatial resolution down to 30 microns and enables noninvasive, *in vivo* visualization of embryonic (E5.5) through to adult mice in real-time, and has been used successfully in detecting mouse embryos [52]. To examine antenatal CAKUT phenotype in *Robo2* embryos, F1 timed pregnant *Robo2* ^del5/flox^ female mice (after positive vaginal plug with *Robo2* ^del5/+^;*Tg* ^Ella-Cre+^ male mice) were anesthetized using inhalant anesthesia of mixed oxygen and anesthetic gas isoflurane (2.5% for anesthesia induction and 1.5% for anesthesia maintenance) through a mouse nose mask tube on the VisualSonics Vevo Compact Anesthesia System (VisualSonics, #SA-11201). Pregnant mice were placed in a supine position on the mouse handling platform with controlled heating, ECG electrodes and temperature monitoring system (VisualSonics, #SA-11436). The mouse heart rate was maintained at 450–500 beats per minute and core body temperature was monitored with a rectal temperature probe coupled to a digital thermometer and was maintained at 37.0°C. The fur of abdominal area was removed using Depilatory cream (Nair, Church & Dwight Co.) and ultrasound transmission gel (Aquasonic 100, Parker Laboratories, NJ) was used as a coupling agent between the skin and the ultrasound transducer. Real-time Micro Visualization (RMV) transducer RMV-708 scanhead (55 MHz center frequency with frequency band between 27.5 MHz to 82.5 MHz, 4.5 mm focal length and axial resolution of 30 µm) was used and mounted on a motorized Vevo integrated rail system III (VisualSonics, #SA-11433) for hand-free serial imaging. Two-dimensional images of individual embryos were obtained in B-mode at a frequency of 30 MHz with an imaging thickness of 40 µm. Grayscale values of ultrasound imaging were calculated by Vevo 770 analytic software for defined areas as quantitative tissue echogenicities to identify and distinguish anatomical structures (e.g. renal pelvis, renal parenchyma, urinary bladder, stomach, spine, etc). The position of most embryos inside the pregnant female body is lateral decubitus. Because the *in vivo* embryo position could not be adjusted inside the uterus, we used easily visible fluid-filled embryonic stomach as a hallmark structure to identify the left side of the body. The enlarged echoless region of renal pelvis (i.e. hydronephrosis) or the ureter (i.e. ureterectasis) could be detected inside *Robo2* mouse embryos by B-mode ultrasound imaging after E17.5 days. Duplex kidney was usually identified under B-mode ultrasound imaging as a combined structure with an echoless hydronephrotic upper pole kidney fused with a normal lower pole kidney with undilated renal pelvis.

### Micro-ultrasound analysis of postnatal CAKUT phenotype in newborn and adult mice

The same Vevo 770 micro-ultrasound imaging system and Vevo Compact Anesthesia System used for pregnant mice above were used for mice after birth. For newborn mice, a special small head mask was used to introduce the same inhalant anesthesia of mixed oxygen and isoflurane. We used the same ultrasound transducer RMV-708 scanhead on newborn, one week and two weeks old mice. Another ultrasound transducer RMV-704 scanhead (40 MHz center frequency with frequency band between 20 MHz to 60 MHz, 6 mm focal length and axial resolution of 40 µm) was used on mice after two weeks old. The normal kidney structure and CAKUT phenotype were detected relatively easily under ultrasound B-mode imaging in mice after birth because we could adjust the position of anesthetized mouse body to allow the best view of renal pelvis and adjacent structures. The largest anteroposterior renal pelvic diameter of each mouse was obtained by Vevo 770 analytic software line measurement tool. Two-dimensional videos of mouse urinary tract and bladder were recorded in B-mode during a 300-frame image recording phase (also called cine loop in Vevo 770 analytic software). Three-dimensional video was obtained from serial transverse 2D images along the entire mouse urinary tract in the caudal-cephalic direction (i.e. ultrasound imaging began from the bladder, moving upper to the ureter, and finally reaching the kidney region), followed by reassembly using Vevo 770 analytic software.

### Micro-ultrasound detection of high-grade dilating VUR in CAKUT mice by urethral catheterization and microbubble ultrasound contrast agents

A polyurethane FunnelCath mouse catheter (Instech Solomon, PA, # PUFC-C30-10) was used for urethral catheterization in adult female mice. This FunnelCath urethral catheter (tapers from 3Fr proximal end to 1.2Fr distal end) has a distal thin segment of 1.2 French gauge (ID: 0.23 mm; OD: 0.41 mm) which can easily enter the external orifice of mouse urethra without injury to the urethral inner epithelium lining. This catheter also has a proximal thick segment of 3 French gauge (ID: 0.66 mm; OD: 1.07 mm) with a Luer-Taper end which can be connected to a syringe or pump for controlled ultrasound contrast agents (or methylene blue) infusion. After mice were anesthetized, the external orifice of urethra was sterilized with povidone-iodine. The distal thin end of FunnelCath mouse catheter was lubricated with sterile mineral oil and inserted gently into the external urethra orifice. The catheter was advanced slowly inside urethra without force until it reached the bladder when urine was drained and visible inside the catheter. The catheter was typed down on the mouse handling platform before the proximal thick end was connected to a syringe filled with microbubble ultrasound contrast agents.

Microbubbles are gas filled small bubbles (∼1–10 µm in diameter) encapsulated with a solid shell [53]. They have been widely used as contract agents for ultrasound imaging in clinical medicine [54] including diagnosis of VUR in children [42], [55]. Option (Perflutren Protein-Type A Microspheres Injectable Suspension, USP) is a FDA-approved ultrasound microbubble contract agent (GE Healthcare, NDC 0407-2707-03). The microbubbles in Option suspension have an albumin shell and perflutren gas core with mean diameter ranging 3.0–4.5 µm and have been used for many indicated ultrasound imaging procedures [56]. Each mL of Optison contains 5.0–8.0×10^8^ microbubbles. We diluted the Option microbubbles 20 fold to 2.5–4.0×10^7^/ml with 1×PBS for retrograde urosonography to evaluate VUR in mice. A syringe infusion pump (Harvard Apparatus, Holliston, MA) was used to infuse diluted microbubbles into the mouse bladder through the urethra catheter at a perfusion rate of 100 µl/min. Coupled with 1.2 French gauge thin urethral catheter, this low speed infusion rate would infuse microbubbles into the bladder without increasing the bladder pressure significantly. Overflow microbubbles will leak out from mouse bladder through external urethra orifice without inducing artificial VUR in wild-type control mice (Fig. 4H).

To detect urinary reflux in *Robo2* mice, Vevo 770 micro-ultrasound system was operated under the control of Vevo Contrast Mode software (VisualSonics, # VS-11639). The RMV-704 scanhead was used to detect microbubble flow movement in the urinary tract before and after contrast agent bladder infusion to detect any retrograde urine flow in the ureter and renal pelvis. In order to visualize urine flow marked by microbubble contrast agents, two ultrasound imaging serial videos were captured under Vevo Contrast Mode software: the first baseline background imaging serial video for the mouse urinary tract before microbubble infusion and a second microbubble enhanced imaging serial video of the same mouse urinary tract after microbubble bladder infusion. Each ultrasound imaging serial video contains 800 individual ultrasound image frame data acquired and stored digitally as a cine loop in Vevo 770 system memory. A final contrast-mode imaging serial video of mouse urinary tract was then digitally constructed by overlaying and comparing baseline background data acquired before microbubble infusion and microbubble enhanced data acquired after microbubble bladder infusion. The appearance and movement of microbubble signal inside the mouse ureter or renal pelvis after microbubble bladder infusion indicated VUR. The microbubble signal could be rendered in either gray-scale (Figure S5, Figure S6, Video S1, and Video S4) or in green-scale (Fig. 4A–H, Video S2, Video S3). Some tissue with strong echogenicity might also be marked as green in the green-scale setting without a real microbubble signal (Fig. 4A–D). These false green signals could be subtracted when compared with baseline background data. A microbubble method assessing low-grade non-dilating VUR in inbred wild-type mice has been described elsewhere [29].

### Confirmation of VUR in *Robo2* mutant mice by methylene blue cystogram

VUR in *Robo2* mutant and control mice were confirmed according to previously published methods [57], [58]. A 27-gauge needle attached via tubing to a syringe filled with 10% methylene blue in saline was inserted into the mouse bladder. The syringe was raised vertically from 30 to 150 cm in relation to the position of the mouse at a rate of 30 cm/min. The hydrostatic pressure (cm-H_2_O) at which micturition or reflux occurs was recorded. The experiment was stopped when there was no reflux at 150 cm for 10 second. VUR confirmation was also performed by infusing 10% methylene blue into the mouse bladder through a mouse urethra catheter at a perfusion rate of 100 µl/min using a Harvard Apparatus syringe infusion pump. Overflow methylene blue leaked out from the mouse bladder through the external urethra orifice without VUR in wild-type control mice.

### Ultrasound-guided percutaneous needle aspiration treatment of severe hydronephrosis in mice

Adult mice with unilateral hydronephrosis were anesthetized with isoflurane on the VisualSonics Vevo Compact Anesthesia System. Dilated renal pelvis was located with Vevo 770 system and the RMV-704 scanhead. Syringe with 30 gauge fine needle was used to quick penetrate abdominal skin and reach to the dilated renal pelvis under the guidance of ultrasound scan. After the needle tip was visible inside the renal pelvis, urine was withdrawn through the syringe until the hydronephrosis disappeared. The needle was then quickly withdrawn out of the skin.

### Histology analysis of mouse embryos, kidneys, and ureterovesical junctions

The mouse embryos bodies, mouse kidneys, or ureterovesical junction regions were dissected and fixed in 4% paraformaldehyde overnight, and taken through a graded alcohol series for paraffin embedding. The paraffin blocks were sectioned at 4 µm using a MT-920 microtome (MICROM) and stained with standard Hematoxylin/Eosin method. The histology slides were evaluated under an Olympus BHT light microscope equipped with a SPOT digital camera system.

### Tissue *in situ* hybridization

*In situ* hybridization analysis was performed with digoxigenin-labeled *Robo2* and *Slit2* riboprobes as previously described [18], [59]. The *Robo2* and *Slit2* cDNA were linearized with *Not*I and probes were generated using the DIG DNA labeling and detection kit (Roche Applied Science). Hybridizations were performed on 4% paraformaldehyde (PFA) fixed OCT embedded mouse embryonic urinary tract frozen sections. Briefly, mouse embryos were collected and dissected in ice-cold PBS and fixed overnight at 4°C in 4% PFA in 0.1 M phosphate buffer and embedded in OCT. Frozen sections were cut on Cryostat and air-dried for 1–3 hours, and fixed again in 4% PFA for 10 minutes at room temperature. After washing with PBS three times, sections were treated with proteinase K (1 mg/ml for 3 minutes at room temperature), washed three times with PBS, acetylated for 10 minutes at room temperature and washed again with PBS. Prehybridizations were performed for 2 hours at room temperature in a humidified chamber. Hybridizations were performed at 68–72°C overnight in a humidified chamber. Prehybridization and hybridization solution were made as following: 50% formamide, 5× SSC, 5× Denhardts, 250 mg/ml baker's yeast RNA, 500 mg/ml herring sperm DNA. Washes were performed at 72°C in 5× SSC for 5–10 minutes, then at 72°C in 0.2× SSC for 1 hour. Sections were stained overnight with anti-digoxigenin antibody (Roche Applied Science) at a 1∶5000 dilution in buffer with 0.1 M Tris-HCl, pH 7.5, 0.15 M NaCl, and 1% heat-inactivated goat serum. After staining at 4°C overnight in a humidified chamber, slides were washed in solution with 0.1 M Tris-HCl, pH 7.5, 0.15 M NaCl. Alkaline phosphatase activity was then detected by developing slides in BCIP, NBT (Roche Applied Science) and 0.25 mg/ml levamisole in a humidified chamber for one day in the dark. Sections were dehydrated and mounted in Permount (Fisher Scientific) and photographed under microscope.

### Early mouse embryonic kidney organ culture and cytokeratin straining

Mouse metanephric embryonic kidneys were mechanically dissected from E11.5 *Robo2* ^del5/del5^ homozygous and control embryos as previously described [60]. Briefly, embryonic kidney tissues were dissected from E11.5 mouse embryos in ice-cold CO2-independent DMEM medium (Invitrogen) or Dulbecco's PBS (Ph 7.2–7.4, with Ca^2+^ and Mg^2+^) containing 10% fetal bovine serum (Invitrogen) using two pairs of fine-tip forceps under dissecting microscope. The isolated embryonic kidney explants were transferred to 12-well Transwell tissue culture plates (Costar #3401) and were placed at the medium-air interface of Transwell polycarbonate membrane filter (0.4 µm pore size). Each individual well within the 12-well Transwell tissue culture plate contains 800 µl DMEM/F12 culture medium (Invitrogen). The kidney explants were cultured for 24 hours at 37°C in tissue culture incubator with 5% CO2 and then fixed in 100% methanol at −20°C for 15–30 minutes. The fixed kidney tissues were washed in 1× PBST for 5 minutes and incubated with anti-pan-cytokeratin primary antibody (Sigma, #C9687, 1∶50 dilution) at 37°C for 1–2 hours. The tissues were then washed in 1× PBS for 5 minutes at room temperature and strained with Cy3 conjugated secondary antibody (Jackson ImmunoResearch, #715-165-150) in a 1∶50 dilution at 37°C for 1 hour. The stained kidney explants were washed in 1× PBS and mounted in VECTASHIELD mounting medium (Vector Laboratories, #H-1000). Ureteric buds were examined and photographed using a Perkin Elmer UltraView LCI multi-point spinning disc laser-scanning confocal microscope.

### Dual and triple immunofluorescence staining of mouse embryonic ureter

Dual and triple immunofluorescence staining were performed on E14.5 *Robo2* mouse embryonic ureter tissues which were dissected and fixed in 4% paraformaldehyde (PFA) at 4°C for 2 hours. The tissues were washed with 1× PBS and followed by two 30-minute incubation in 15% and 30% sucrose for cryoprotection. The fixed embryonic ureters were frozen embedded in OCT compound and sectioned at ∼8 µm thickness using Cryostat. Sections were washed in 1× PBS buffer and incubated in 1× casein blocking solution (Vector Laboratories, #SP-5020) for 30 minutes at room temperature. Sections were then stained with a monoclonal anti-α smooth muscle actin (anti-αSMA) antibody (clone 1A4, Sigma-Aldrich, # A5228) at 1∶200 dilution in 1× casein blocking solution at 4°C overnight. For double staining with E-cadherin, a goat-anti-mouse E-cadherin primary antibody (R&D Systems, #AF748) was also added to the sections at 1∶100 dilution. After three times 5-minute washes with 1× PBS buffer, sections were incubated in a Cy3 conjugated secondary antibody (Chemicon, #AP192C, 1∶400 dilution) and a FITC conjugated secondary antibody (Jackson ImmunoResearch, #705-095-003, 1∶100 dilution) for 4 hours at 4°C followed by 15 minutes at room temperature. For lectin staining, the ureter sections were incubated with FITC-conjugated urothelium marker lectin *Dolichos biflorus* (DBA, Sigma-Aldrich, #L9142) 4 hours at 4°C followed by 15 minutes at room temperature. After three times 5-minute washes in 1× PBS, dual-stained sections (anti-αSMA and FITC-conjugated DBA) were mounted with regular VECTASHIELD mounting medium (Vector Laboratories, #H-1000) and triple-stained sections (anti-αSMA plus anti-E-cadherin) were mounted with modified VECTASHIELD mounting medium with DAPI (Vector Laboratories, #H-1200). Stained sections were examined and photographed using a Perkin Elmer UltraView LCI multi-point spinning disc laser-scanning confocal microscope.

### Statistics and mouse survival curve analysis

The renal pelvic diameters in *Robo2* mice with progressive hydronephrosis were analyzed with Student's *t*-test. The survival curve of *Robo2* mice with antenatal hydronephrosis was analyzed with NCSS 2007 software (Kaysville, Utah).

## Supporting Information

Figure S1 **Mouse fetal kidneys can be detected by transabdominal ultrasonography at E15.5.**
(PDF)

Figure S2 **Breeding scheme to generate** ***Robo2*** **^del5/del5^ homozygotes,** ***Robo2*** **^del5/del5^↔** ***Robo2*** **^del5/flox^ mosaic mutant mice, and** ***Robo2*** **^flox/+^ controls.**
(PDF)

Figure S3 **Antenatal congenital urinary tract defects in the same** ***Robo2*** **mutant mice can be monitored and followed from prenatal to postnatal stages.**
(PDF)

Figure S4 **Spontaneous regression of hydronephrosis in a** ***Robo2*** **mosaic mouse with duplex kidney.**
(PDF)

Figure S5 **Urethral catheter inside a mouse bladder can be detected by urosonography.**
(PDF)

Figure S6 **Microbubble ultrasound contrast agents (UCA) were washed out from the renal pelvis shortly after reflux in a** ***Robo2*** **mosaic mouse.**
(PDF)

Figure S7 ***Slit2*** **is expressed in the developing mouse UVJ region.**
(PDF)

Figure S8 **Ureteral smooth muscle defect in a** ***Robo2*** **homozygous embryo at lower magnification.**
(PDF)

Table S1 **Sensitivity and specificity of antenatal hydronephrosis detection in E18.5** ***Robo2*** **mutant mouse embryos by micro-ultrasonography.**
(PDF)

Table S2 **Sensitivity and specificity of hydronephrosis detection in** ***Robo2*** **mutant newborn mice by micro-ultrasonography.**
(PDF)

Table S3 **Survival of 29** ***Robo2*** **mutant mice with antenatal hydronephrosis (25 bilateral; 4 unilateral) in the first 6 weeks after birth.**
(PDF)

Table S4 **Largest anteroposterior renal pelvic diameter (mm) measured at different stages from prenatal E18.5 to postnatal week-6 in seven survived** ***Robo2*** **mosaic mice with antenatal hydronephrosis (2 bilateral; 5 unilateral – total 9 kidney units).**
(PDF)

Video S1 **Infusion of microbubble ultrasound contrast agents into the bladder of a 6-week old** ***Robo2*** **mouse through a urethra catheter in gray-scale.**
(WMV)

Video S2 **Infusion of microbubble ultrasound contrast agents in green-scale into the bladder of a 6-week old** ***Robo2*** **mouse under contrast imaging mode.**
(WMV)

Video S3 **Retrograde flow of microbubble ultrasound contrast agents in green-scale refluxing into dilated renal pelvis from the bladder of a** ***Robo2*** **mosaic mouse under contrast imaging mode.**
(WMV)

Video S4 **Microbubble ultrasound contrast agents (UCA) washout from renal pelvis shortly after reflux in a** ***Robo2*** **mosaic mouse under B-mode ultrasound in gray-scale.**
(WMV)

Video S5 **Ultrasound-guided percutaneous needle insertion into the renal pelvis of a** ***Robo2*** **mosaic mouse with hydronephrosis.**
(WMV)

Video S6 **Ultrasound-guided percutaneous needle aspiration of urine from dilated renal pelvis of a** ***Robo2*** **mosaic mouse with hydronephrosis.** Noting the collapse of the renal pelvis after ultrasound-guided needle aspiration.
(WMV)

Video S7 **Two-dimensional B-mode ultrasound video of the entire urinary tract structure in a** ***Robo2*** **newborn mosaic mouse in the caudal-cephalic scanning direction (i.e. ultrasound imaging began with the bladder, then golf-hole like enlarged ureterovesical junction, moving upper to the dilated ureter, and finally showing the renal pelvis region with hydronephrosis).**
(WMV)

Video S8 **Three-dimensional reassembly of the entire urinary tract structure using 2D images captured in video S7 from the same** ***Robo2*** **newborn mosaic mouse with golf-hole like enlarged ureterovesical junction, megaureter and hydronephrosis.**
(WMV)
