# Supplementary material for: Noninvasive Assessment of Antenatal Hydronephrosis in Mice Reveals a Critical Role for Robo2 in Maintaining Anti-Reflux Mechanism
Source: PLoS One. 2011 Sep 20;6(9):e24763. doi: 10.1371/journal.pone.0024763 (PMC3176762; doi:10.1371/journal.pone.0024763)
Supplement: Figure S1 — Mouse fetal kidneys can be detected by transabdominal ultrasonography at E15.5. (PDF) [file pone.0024763.s001.pdf]

Figure S1

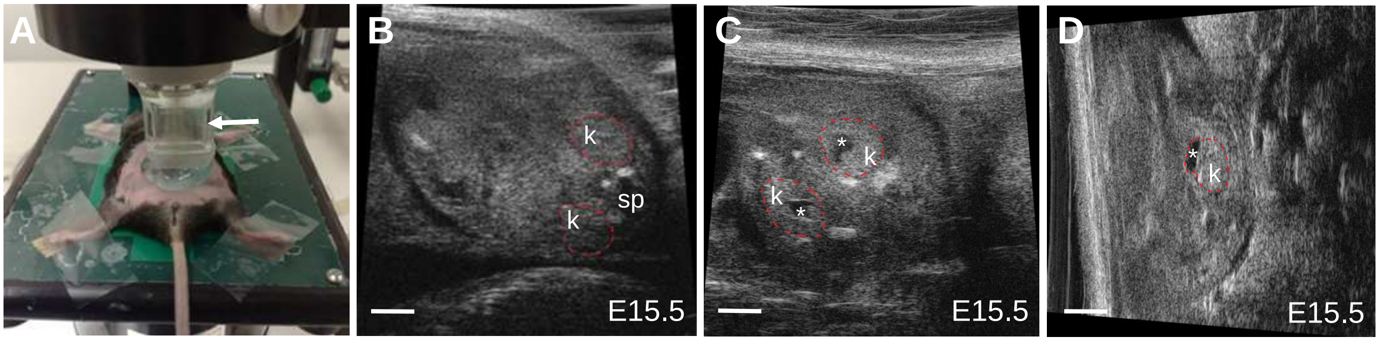

Figure S1. Mouse fetal kidneys were detected by transabdominal ultrasonography at E15.5. (**A**) Ultrasound scanhead position (white arrow) on an E15.5 pregnant female mouse under continuous isoflurane anesthesia. (**B**,**C**) Ultrasonographic transverse planes depicting normal E15.5 fetal kidney (k); sp, spine; renal pelvis (asterisks in C) was visible in some normal E15.5 fetal kidney. (**D**) An ultrasonographic coronal plane depicting renal pelvic dilatation (asterisk in D) in an E15.5 kidney (k) which was confirmed as duplex kidney at birth. The outline of the kidneys depicted in ultrasound images were marked by red dot line. Scale bars, 1.0 mm.
