# Supplementary material for: Noninvasive Assessment of Antenatal Hydronephrosis in Mice Reveals a Critical Role for Robo2 in Maintaining Anti-Reflux Mechanism
Source: PLoS One. 2011 Sep 20;6(9):e24763. doi: 10.1371/journal.pone.0024763 (PMC3176762; doi:10.1371/journal.pone.0024763)
Supplement: Figure S2 — Breeding scheme to generate Robo2 del5/del5 homozygotes, Robo2 del5/del5↔ Robo2 del5/flox mosaic mutant mice, and Robo2 flox/+ controls. (PDF) [file pone.0024763.s002.pdf]

Figure S2

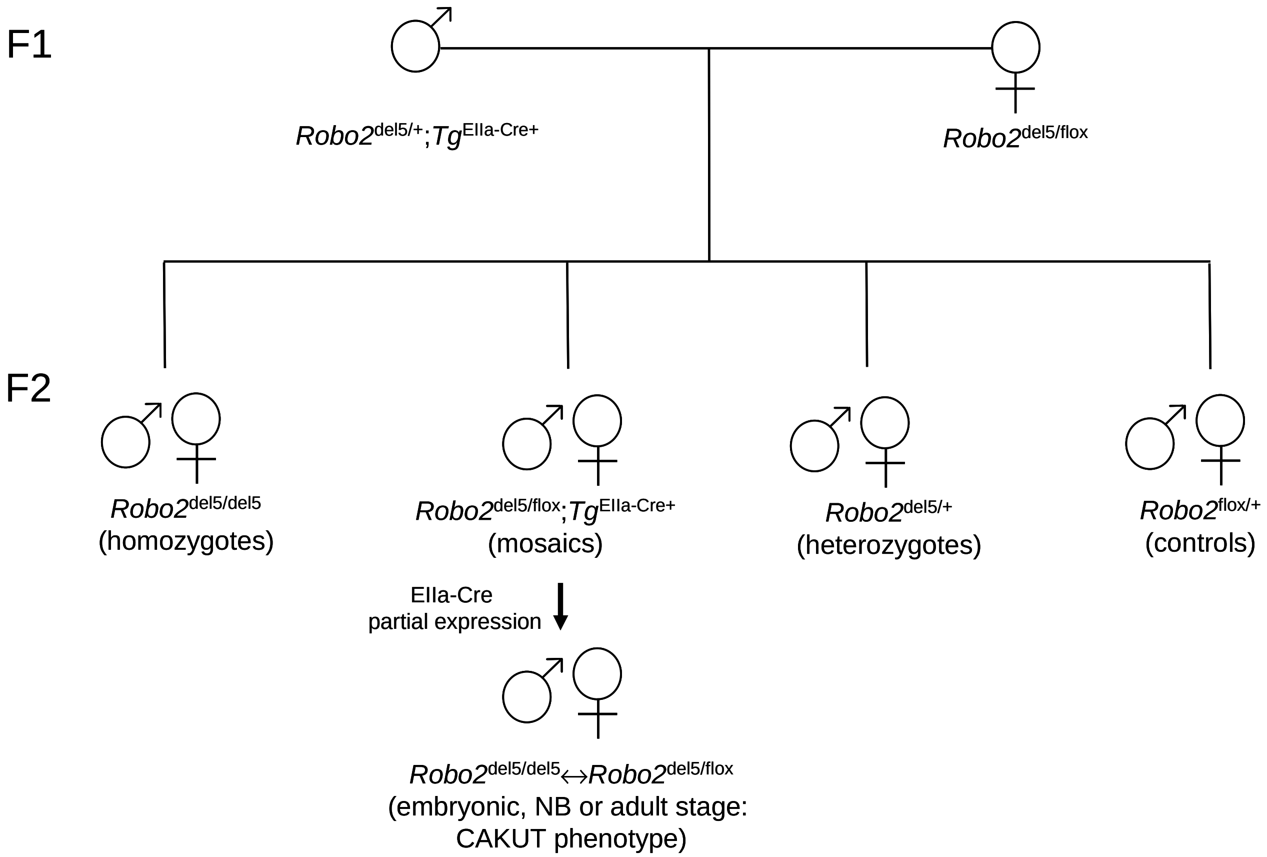

Figure S2. Breeding scheme: *Robo2* heterozygous mating to generate *Robo2*$^{del5/del5}$ homozygous mice with 100% CAKUT phenotype, *Robo2*$^{del5/del5}$↔*Robo2*$^{del5/flox}$ mosaic mutant mice with ~70% CAKUT phenotype. *Robo2*$^{flox/+}$ mice with no CAKUT phenotype were used as controls.
