# Supplementary material for: Noninvasive Assessment of Antenatal Hydronephrosis in Mice Reveals a Critical Role for Robo2 in Maintaining Anti-Reflux Mechanism
Source: PLoS One. 2011 Sep 20;6(9):e24763. doi: 10.1371/journal.pone.0024763 (PMC3176762; doi:10.1371/journal.pone.0024763)
Supplement: Figure S3 — Antenatal congenital urinary tract defects in the same Robo2 mutant mice can be monitored and followed from prenatal to postnatal stages. (PDF) [file pone.0024763.s003.pdf]

Figure S3

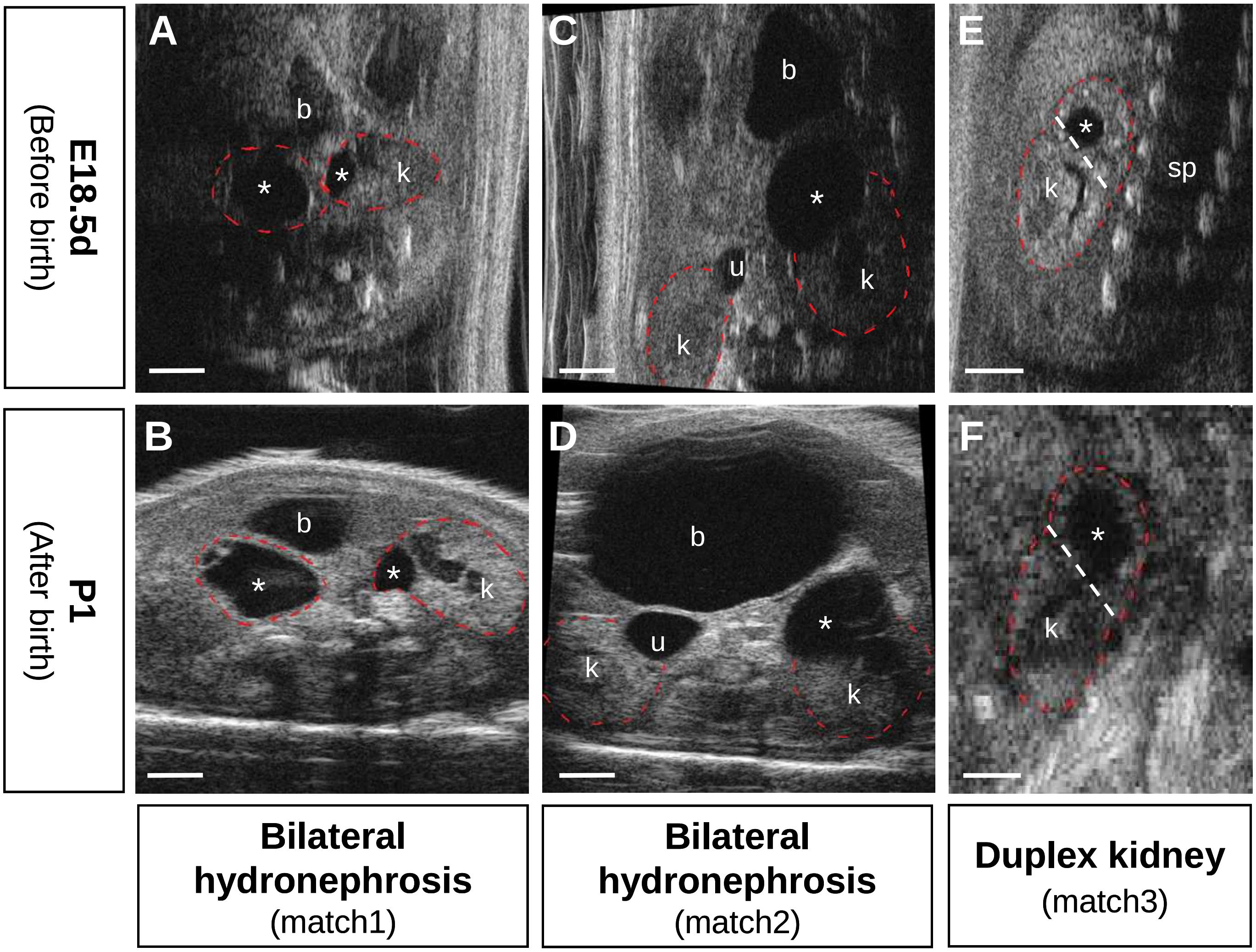

Figure S3. Antenatal congenital urinary tract defects in *Robo2* mutant mice can be monitored and followed from prenatal to postnatal stages. Upper panels (**A**,**C**,**E**) showing ultrasonographic images from E18.5 *Robo2* embryos before birth. Lower panels (**B**,**D**,**F**) showing ultrasonographic images from corresponding postnatal day-1 (P1) *Robo2* mice after birth. Left column (**A**,**B**) depicting follow-up and match of bilateral hydronephrosis (asterisks) phenotype in a *Robo2* mosaic mutant from prenatal to postnatal stages (Match 1). Middle column (**C**,**D**) depicting follow-up and match of left hydroureter (u) and right hydronephrosis (asterisks) phenotype in a second *Robo2* mosaic mutant from prenatal to postnatal stages (Match 2). Right column (**E**,**F**) depicting follow-up and match of a duplex kidney phenotype with upper pole hydronephrosis (asterisk) in a third *Robo2* mosaic mutant from prenatal to postnatal stages (Match 3); upper and lower pole of duplex kidney were separated by an artificial white dot line. Scale bars, 1.0 mm. Abbreviation: b, urinary bladder; k, kidney; sp, spine; u, ureter.
