# Supplementary material for: Noninvasive Assessment of Antenatal Hydronephrosis in Mice Reveals a Critical Role for Robo2 in Maintaining Anti-Reflux Mechanism
Source: PLoS One. 2011 Sep 20;6(9):e24763. doi: 10.1371/journal.pone.0024763 (PMC3176762; doi:10.1371/journal.pone.0024763)
Supplement: Figure S4 — Spontaneous regression of hydronephrosis in a Robo2 mosaic mouse with duplex kidney. (PDF) [file pone.0024763.s004.pdf]

Figure S4

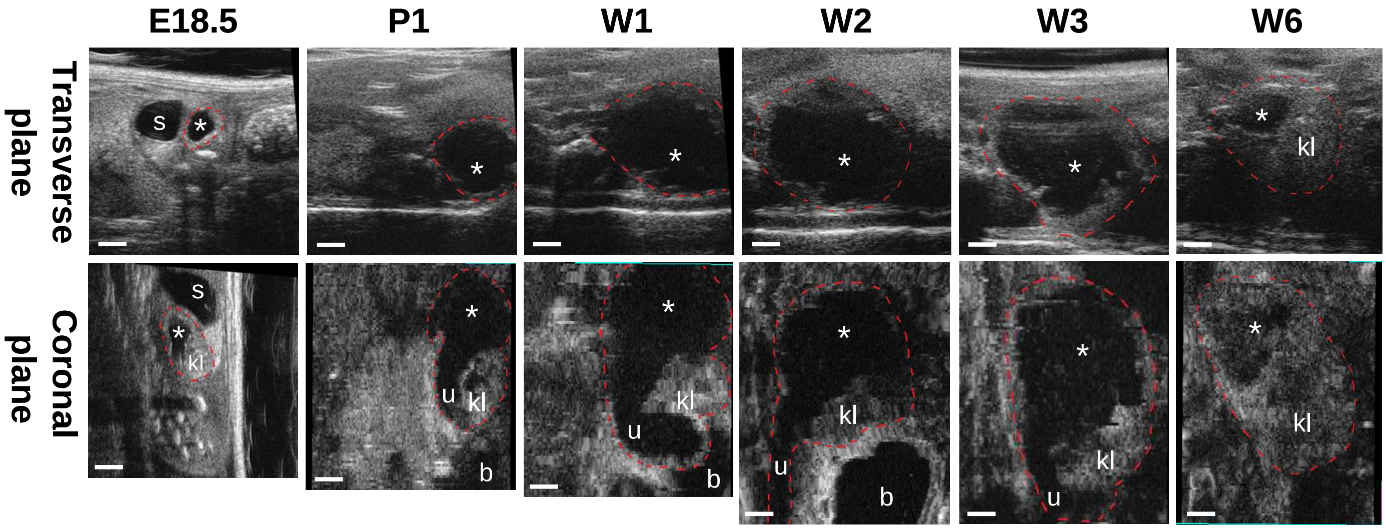

Figure S4. Spontaneous regression of hydronephrosis in *Robo2* mosaic mutant mice with duplex kidney. Upper figure panels: transverse plane ultrasound imaging of upper pole hydronephrosis (asterisks) progression in a *Robo2* mosaic mutant mouse with duplex kidney from embryonic E18.5 to postnatal 6-week old (W6) showing hydronephrosis reached peak at 1-2 weeks (W1, W2) and spontaneous regressed at 6 weeks. Lower figure panels: coronal plane ultrasound imaging of the same *Robo2* mosaic mouse with duplex kidney and upper pole hydronephrosis (asterisks); Scale bars, 1.0 mm. Abbreviation: b, bladder; kl, lower pole of duplex kidney without hydronephrosis; s, stomach; u, ureter; P1: postnatal day-1; W1 to W6: week-1 to week-6.
