# Supplementary material for: Noninvasive Assessment of Antenatal Hydronephrosis in Mice Reveals a Critical Role for Robo2 in Maintaining Anti-Reflux Mechanism
Source: PLoS One. 2011 Sep 20;6(9):e24763. doi: 10.1371/journal.pone.0024763 (PMC3176762; doi:10.1371/journal.pone.0024763)
Supplement: Figure S5 — Urethral catheter inside a mouse bladder can be detected by urosonography. (PDF) [file pone.0024763.s005.pdf]

Figure S5

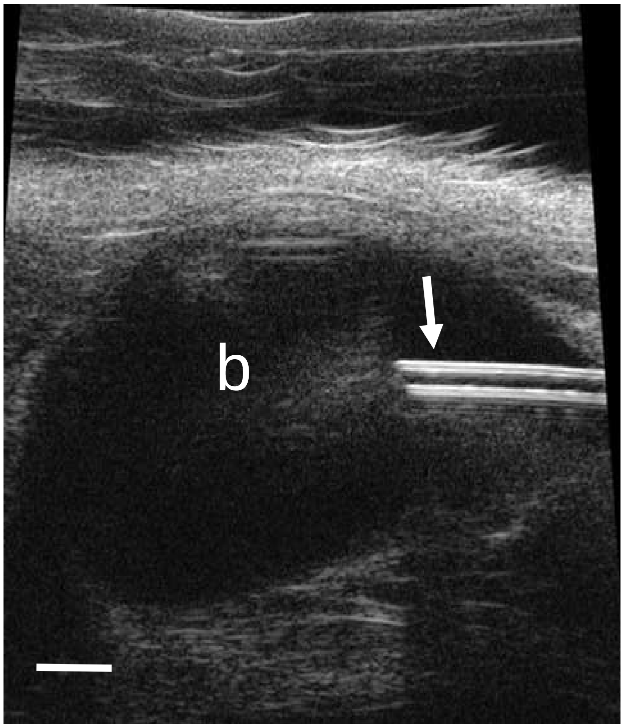

Mouse urethral catheterization

Figure S5. Urethral catheter inside a mouse bladder can be detected by urosonography. Urethral catheter (white arrow) was visible inside a 6-week old female mouse bladder (b) under B-mode ultrasound scan. Scale bars, 1.0 mm.
