# Supplementary material for: Noninvasive Assessment of Antenatal Hydronephrosis in Mice Reveals a Critical Role for Robo2 in Maintaining Anti-Reflux Mechanism
Source: PLoS One. 2011 Sep 20;6(9):e24763. doi: 10.1371/journal.pone.0024763 (PMC3176762; doi:10.1371/journal.pone.0024763)
Supplement: Figure S6 — Microbubble ultrasound contrast agents (UCA) were washed out from the renal pelvis shortly after reflux in a Robo2 mosaic mouse. (PDF) [file pone.0024763.s006.pdf]

Figure S6

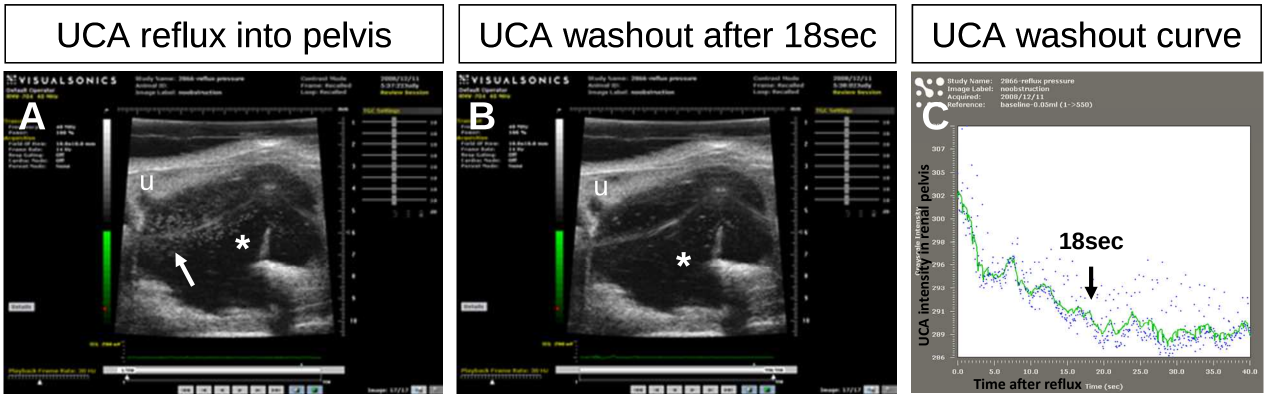

Figure S6. Microbubble ultrasound contrast agents (UCA) were washed out from renal pelvis shortly after reflux in *Robo2* mutant mice. (**A**) Ultrasound contrast agents (UCA, arrow) refluxed into the renal pelvis (asterisk) through the ureter (u) after bladder infusion. (**B**) UCA disappeared from the renal pelvis (asterisk) through the ureter (u) 18 second after reflux, suggesting no urinary tract obstruction. (**C**) UCA washout curve showing microbubble flowed out of the renal pelvis 18 second after it appeared.
