# Supplementary material for: Noninvasive Assessment of Antenatal Hydronephrosis in Mice Reveals a Critical Role for Robo2 in Maintaining Anti-Reflux Mechanism
Source: PLoS One. 2011 Sep 20;6(9):e24763. doi: 10.1371/journal.pone.0024763 (PMC3176762; doi:10.1371/journal.pone.0024763)
Supplement: Figure S7 — Slit2 is expressed in the developing mouse UVJ region. (PDF) [file pone.0024763.s007.pdf]

Figure S7

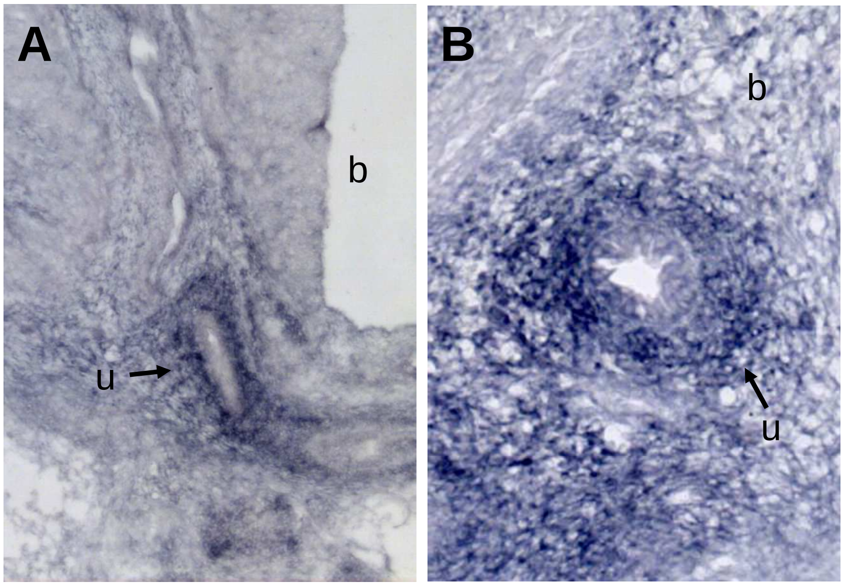

Figure S7. *Slit2* is expressed in the developing mouse UVJ region. (**A**) *In situ* hybridization analysis shows that *Slit2* is expressed in the distal ureter (u, arrow) inside the bladder (b) wall of an E17.5 mouse embryo. (**B**) Higher magnification of the UVJ region in an E15.5 mouse embryo showing strong *Slit2* expression around the developing distal ureter (u, arrow) inside the bladder (b) wall.
