# Supplementary material for: Noninvasive Assessment of Antenatal Hydronephrosis in Mice Reveals a Critical Role for Robo2 in Maintaining Anti-Reflux Mechanism
Source: PLoS One. 2011 Sep 20;6(9):e24763. doi: 10.1371/journal.pone.0024763 (PMC3176762; doi:10.1371/journal.pone.0024763)
Supplement: Figure S8 — Ureteral smooth muscle defect in a Robo2 homozygous embryo at lower magnification. (PDF) [file pone.0024763.s008.pdf]

Figure S8

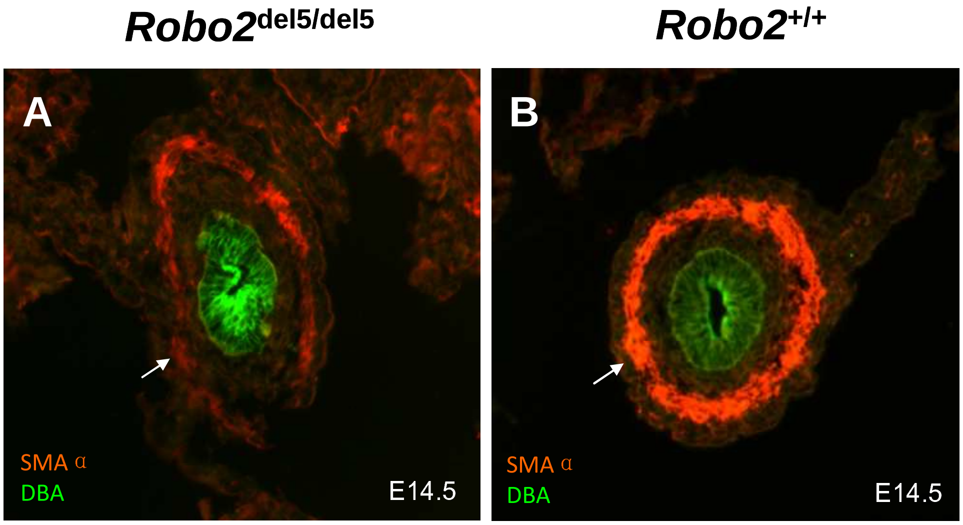

Figure S8. Disarrangement and reduced abundance of ureteral smooth muscle in E14.5 *Robo2*$^{del5/del5}$ homozygous embryos at low magnification. Anti-smooth muscle α actin antibody (SMAα, red) showing reduced and irregular ureteral smooth muscle staining (arrow in **A**) in an E14.5 *Robo2*$^{del5/del5}$ homozygous embryo compared with its wild-type littermate control (arrow in **B**). The urothelium (stained by lection DBA, green) appeared normal in both *Robo2*$^{del5/del5}$ homozygous and wild-type controls; magnification: 100x.
