# Supplementary material for: Noninvasive Assessment of Antenatal Hydronephrosis in Mice Reveals a Critical Role for Robo2 in Maintaining Anti-Reflux Mechanism
Source: PLoS One. 2011 Sep 20;6(9):e24763. doi: 10.1371/journal.pone.0024763 (PMC3176762; doi:10.1371/journal.pone.0024763)
Supplement: Table S1 — Sensitivity and specificity of antenatal hydronephrosis detection in E18.5 Robo2 mutant mouse embryos by micro-ultrasonography. (PDF) [file pone.0024763.s009.pdf]

Table S1

Sensitivity and specificity of hydronephrosis detection in E18.5 *Robo2* mouse embryos by micro-ultrasonography

		**Histology** (standard)	
		Hydronephrosis (Positive)	Normal (Negative)
**Ultrasound** (test outcome)	Hydronephrosis (Positive)	11 (true positive)	2 (false positive)
	Normal & Not detected (Negative)	2 (false negative)	14 (true negative)

Four pregnant *Robo2*$^{del5/flox}$ mice at E18.5 gestation were subjected to ultrasound scanning to detect antenatal hydronephrosis in E18.5 embryos. The embryos were then dissected and analyzed by histology. 27 embryos were detected by ultrasonography while actual 29 embryos were found with dissection.
