# Supplementary material for: Noninvasive Assessment of Antenatal Hydronephrosis in Mice Reveals a Critical Role for Robo2 in Maintaining Anti-Reflux Mechanism
Source: PLoS One. 2011 Sep 20;6(9):e24763. doi: 10.1371/journal.pone.0024763 (PMC3176762; doi:10.1371/journal.pone.0024763)
Supplement: Table S2 — Sensitivity and specificity of hydronephrosis detection in Robo2 mutant newborn mice by micro-ultrasonography. (PDF) [file pone.0024763.s010.pdf]

Table S2

Sensitivity and specificity of hydronephrosis detection in *Robo2* newborn mice by micro-ultrasonography

		**Histology** (standard)	
		Hydronephrosis (Positive)	Normal (Negative)
**Ultrasound** (test outcome)	Hydronephrosis (Positive)	21 (true positive)	0 (false positive)
	Normal & Not detected (Negative)	0 (false negative)	25 (true negative)

46 newborn mice from *Robo2* heterozygous mating were subjected to ultrasound scanning to detect postnatal hydronephrosis. The newborn mice were then dissected and analyzed by histology.
