# Supplementary material for: Noninvasive Assessment of Antenatal Hydronephrosis in Mice Reveals a Critical Role for Robo2 in Maintaining Anti-Reflux Mechanism
Source: PLoS One. 2011 Sep 20;6(9):e24763. doi: 10.1371/journal.pone.0024763 (PMC3176762; doi:10.1371/journal.pone.0024763)
Supplement: Table S3 — Survival of 29 Robo2 mutant mice with antenatal hydronephrosis (25 bilateral; 4 unilateral) in the first 6 weeks after birth. (PDF) [file pone.0024763.s011.pdf]

Table S3

Survival of 29 *Robo2* mutant mice with antenatal hydronephrosis (25 bilateral; 4 unilateral) in the first 6 weeks after birth

	**Survival at 48hrs (total)**	**Survival at 1w (total)**	**Survival at 2w (total)**	**Survival at 3w (total)**	**Survival at 6w (total)**
**Bilateral antenatal hydronephrosis**	5 (25)	4 (25)	3 (25)	3 (25)	2 (25)
**Unilateral antenatal hydronephrosis**	4 (4)	4 (4)	4 (4)	4 (4)	4 (4)
**Control without antenatal hydronephrosis**	30 (30)	30 (30)	30 (30)	30 (30)	30 (30)

Abbreviations: hrs: hours; w: weeks
