# Supplementary material for: Noninvasive Assessment of Antenatal Hydronephrosis in Mice Reveals a Critical Role for Robo2 in Maintaining Anti-Reflux Mechanism
Source: PLoS One. 2011 Sep 20;6(9):e24763. doi: 10.1371/journal.pone.0024763 (PMC3176762; doi:10.1371/journal.pone.0024763)
Supplement: Table S4 — Largest anteroposterior renal pelvic diameter (mm) measured at different stages from prenatal E18.5 to postnatal week-6 in seven survived Robo2 mosaic mice with antenatal hydronephrosis (2 bilateral; 5 unilateral – total 9 kidney units). (PDF) [file pone.0024763.s012.pdf]

Table S4

The anteroposterior renal pelvic diameter (mm) measured at different stages from prenatal E18.5 to postnatal week-6 in seven survived *Robo2* mosaic mice with antenatal hydronephrosis (2 bilateral; 5 unilateral – total 9 kidney units):

Kidney ID	E18.5	P1	W1	W2	W3	W6
**1 - left**	1.7	2.13	4.23	5.37	6.25	6.55
**1 - right**	1.01	1.45	2.16	3.24	3.55	7.32
**2 - left**	0.82	1.42	2.17	3.94	6.1	10.31
**2 - right**	1.72	3.17	4.32	5.6	5.27	7.26
**3**	1.54	2.4	2.97	7.98	10.24	7.59
**4**	1.81	2.17	4.38	5.2	7.41	9.37
**5**	1.95	4.15	6.07	8.75	12.3	14.5
**6**	0.79	1.23	2.37	4.1	4.03	7.35
**7**	2.01	2.29	6.66	8.52	11.02	12.1
**Mean diameter (mm)**	1.48	2.27	3.93	5.86	7.35	9.15

Note: E18.5: embryonic day 18.5; P1: postnatal day-1; W1 to W6: week-1 to week-6;
